# The structure of lipopeptides impacts their antiviral activity and mode of action against SARS-CoV-2 *in vitro*

**DOI:** 10.1128/aem.01036-24

**Published:** 2024-10-24

**Authors:** Alexis C. R. Hoste, Willy Smeralda, Aurélien Cugnet, Yves Brostaux, Magali Deleu, Mutien Garigliany, Philippe Jacques

**Affiliations:** 1 MiPI, TERRA Teaching and Research Centre, Joint Research Unit BioEcoAgro, UMRt 1158, Gembloux Agro-Bio Tech, University of Liège, Gembloux, Belgium; 2 Veterinary Pathology, FARAH Research Centre, Faculty of Veterinary Medicine, University of Liège, Liège, Belgium; 3 LBMI, TERRA Teaching and Research Centre, Joint Research Unit BioEcoAgro, UMRt 1158, Gembloux Agro-Bio Tech, University of Liège, Gembloux, Belgium; 4 Applied Statistics, Computer Science and Modelling laboratory, TERRA Teaching and Research Centre, Gembloux Agro-Bio Tech, University of Liège, Gembloux, Belgium; Centers for Disease Control and Prevention, Atlanta, Georgia, USA

**Keywords:** lipopeptides, antiviral, cytotoxicity, SARS-CoV-2, structure-activity relationship, surfactin, WLIP, fengycin, caspofungin

## Abstract

Microbial lipopeptides are synthesized by nonribosomal peptide synthetases and are composed of a hydrophobic fatty acid chain and a hydrophilic peptide moiety. These structurally diverse amphiphilic molecules can interact with biological membranes and possess various biological activities, including antiviral properties. This study aimed to evaluate the cytotoxicity and antiviral activity against severe acute respiratory syndrome coronavirus 2 (SARS-CoV-2) of 15 diverse lipopeptides to understand their structure-activity relationships. Non-ionic lipopeptides were generally more cytotoxic than charged ones, with cationic lipopeptides being less cytotoxic than anionic and non-ionic variants. At 100 µg/mL, six lipopeptides reduced SARS-CoV-2 RNA to undetectable levels in infected Vero E6 cells, while six others achieved a 2.5- to 4.1-log reduction, and three had no significant effect. Surfactin, white line-inducing principle (WLIP), fengycin, and caspofungin emerged as the most promising anti-SARS-CoV-2 agents. Detailed analysis revealed that these four lipopeptides affected various stages of the viral life cycle involving the viral envelope. Surfactin and WLIP significantly reduced viral RNA levels in replication assays, comparable to neutralizing serum. Surfactin uniquely inhibited viral budding, while fengycin impacted viral binding after pre-infection treatment of the cells. Caspofungin demonstrated a lower antiviral effect compared to the others. Key structural traits of lipopeptides influencing their cytotoxic and antiviral activities were identified. Lipopeptides with a high number of amino acids, especially charged (preferentially anionic) amino acids, showed potent anti-SARS-CoV-2 activity. This research paves the way for designing new lipopeptides with low cytotoxicity and high antiviral efficacy, potentially leading to effective treatments.

**IMPORTANCE:**

This study advances our understanding of how lipopeptides, which are molecules mostly produced by bacteria, with both fat and protein components, can be used to fight viruses like severe acute respiratory syndrome coronavirus 2 (SARS-CoV-2). By analyzing 15 different lipopeptides, researchers identified key structural features that make some of these molecules particularly effective at reducing viral levels while being less harmful to cells. Specifically, lipopeptides with certain charged amino acids were found to have the strongest antiviral effects. This research lays the groundwork for developing new antiviral treatments that are both potent against viruses and safe for human cells, offering hope for better therapeutic options in the future.

## INTRODUCTION

Over the past 20 years, three coronaviruses have crossed the species barrier with an evolving virulence and with the potential to cause pandemics, as shown by recombinant severe acute respiratory syndrome (SARS) and Middle East respiratory syndrome (MERS) coronavirus (CoV) (1, 2) and, more recently, by the newly emerged severe acute respiratory syndrome coronavirus 2 (SARS-CoV-2), the causative agent of coronavirus disease 2019 (COVID-19) (3). Vaccines have rapidly been developed (4, 5). However, to further reduce viral transmission and mortality, effective antiviral treatments are still needed. Since the emergence of SARS-CoV-2, more than 700 molecules have been reported to have anti-SARS-CoV-2 activity targeting different stages of the replication cycle of the virus, with only a few of them marketed to treat COVID-19 (6).

Briefly, SARS-CoV-2 virions bind to cellular receptors such as angiotensin-converting enzyme 2 (ACE2) and use transmembrane protease serine 2 for spike priming, which promotes the fusion of viral and cellular membranes (7). After fusion, the genomic RNA is released, and open reading frame 1a (ORF1a) and ORF1b are translated into two polyproteins, pp1a and pp1ab. These are co-translationally and post-translationally processed into the individual non-structural proteins that form the viral replication and transcription complex. The formation of viral replication organelles creates a protective microenvironment for viral genomic RNA replication and the transcription of subgenomic mRNAs comprising the characteristic nested set of coronavirus mRNAs (8, 9). Translated structural proteins translocate into endoplasmic reticulum (ER) membranes and transit through the ER-to-Golgi intermediate compartment, where interaction with N-encapsidated genomic RNA results in budding into the lumen of secretory vesicular compartments (10). Finally, virions undergo lysosomal trafficking for egress (11).

Only nirmatrelvir-ritonavir, remdesivir, molnupiravir, and 11 monoclonal antibodies have been marketed to treat COVID-19 (6). Nirmatrelvir is an inhibitor of the 3C-like protease involved in the cleavage of the SARS-CoV-2 polyprotein. In combination with ritonavir, a strong CYP3A4 inhibitor, it was shown to reduce the severity of the disease and lower the viral load (12). Remdesivir and molnupiravir are inhibitors of RNA-dependent RNA polymerase. While remdesivir has shown mixed results in clinical trials, molnupiravir has raised safety concerns (13–15). Finally, monoclonal antibodies target and block the interaction between the spike protein and the ACE2 receptor. However, the emergence of viral variants may lead to evasion of these antibodies (16). While the previously mentioned molecules have been marketed as treatments for COVID-19, their antiviral effectiveness has certain limitations. Consequently, there is a significant demand for new antiviral compounds targeting various phases of the SARS-CoV-2 replication cycle.

Lipopeptides have been isolated from different genera of bacteria, fungi, and potentially animals (17). These compounds are secondary metabolites synthesized by multienzymatic proteins called nonribosomal peptide synthetases (NRPS), assisted by polyketide synthases in some cases. They are composed of a hydrophobic fatty acid (FA) chain and a hydrophilic peptide moiety. The FA chain can vary in length, isomeric form, or saturation and can be β-hydroxylated, β-aminated, or guanylated. The peptide moiety consists of a variable sequence of monomers, including mainly amino acid residues in the L or D form. Lipopeptides are cyclic, partially cyclic, or linear, with FA chain lengths varying from C7 [pelgipeptin (18–20)] to C43 [licheniformin (21)] and peptide moieties varying from 2 amino acids [i.e., serrawettin W1 (22, 23)] to 25 amino acids [syringopeptin 25 (24)]. The peptide moiety can be positively charged, negatively charged, zwitterionic, or non-charged at pH 7 (17).

Due to their amphiphilic structure, lipopeptides can interact with biological membranes. They have been found to have a wide range of biological activities, the most notable of which are antibacterial and antifungal activities. They also have the ability to induce systemic resistance in plants and exhibit cytotoxic and antiviral activities (25). Among the various lipopeptides studied, surfactin, produced by *Bacillus* sp., has shown significant antiviral activity. Surfactin can inactivate a wide range of enveloped DNA and RNA viruses, including transmissible gastroenteritis virus, porcine epidemic diarrhea virus (PEDV), and human coronavirus 229E, MERS, and recombinant SARS-CoV (26–32). Surfactin was found to have two modes of action against viruses: at a high concentration (100 µg/mL), it disrupts virion integrity, and at lower concentrations, it acts as a membrane fusion inhibitor (26, 30, 31). Yuan et al. demonstrated that surfactin could protect piglets against PEDV infection and showed that surfactin analogs obtained by chemical synthesis had antiviral activity similar to that of surfactin but had lower hemolytic activity (31, 32).

Recent studies have explored the potential activity of lipopeptides against SARS-CoV-2. A few studies using *in silico* analysis and molecular docking have explored the interaction potential of lipopeptides with different viral targets (33–35). *In vitro* studies are rare. Crovella et al. showed that surfactin at 1 mg/mL was able to reduce SARS-CoV-2 infectivity in Vero E6 cells (36). Nakajima et al. studied the anti-SARS-CoV-2 activity of echinocandins and reported that they were able to reduce viral RNA levels when they were added at certain stages of viral replication, suggesting that these lipopeptides target viral replication (37). Finally, Shekunov et al. examined the antiviral activity of aculeacin A, anidulafungin, iturin A, and mycosubtilin *in vitro* (38). When co-incubated with SARS-CoV-2, 12.5 µg/mL iturin A and 25 µg/mL mycosubtilin could reduce the viral titer and SARS-CoV-2 cytopathic effects (CPEs) on Vero cells without any apparent cytotoxicity.

The aim of this study was to enhance the understanding of the structure-activity relationships of lipopeptides by identifying the structural features influencing their cytotoxic and antiviral activities. Fifteen representative lipopeptides were selected and screened to assess their impact on Vero E6 cell cytotoxicity and their antiviral potential against SARS-CoV-2 *in vitro*. The number of amino acids, the number of charged amino acids, and the negative charges at pH 7 were the structural traits most correlated with the cytotoxicity and antiviral activity of the lipopeptides. Among the lipopeptides tested, surfactin, white line-inducing principle (WLIP), fengycin, and caspofungin demonstrated the most potent antiviral activity. These lipopeptides were selected for further investigation to elucidate their mode of action against SARS-CoV-2. Our results revealed that these lipopeptides effectively reduce the total viral RNA concentration at non-cytotoxic concentrations and target various stages of the SARS-CoV-2 life cycle, all of which involve the viral envelope. This study represents the first comprehensive evaluation of the anti-SARS-CoV-2 activity of lipopeptides, considering both their cytotoxicity and mode of action. Our results highlight the promising antiviral potential of specific lipopeptides and contribute to a deeper understanding of how the structure of these compounds influences their activity. This work provides valuable insights for the design of novel lipopeptides with low cytotoxicity and high antiviral activity, paving the way for the development of effective treatments.

## MATERIALS AND METHODS

### Virus and cells

*Chlorocebus* sp. kidney epithelial cells (Vero E6; ATCC VERO C1008) were cultured in Dulbecco’s Modified Eagle medium (DMEM) High Glucose w/ Stable Glutamine w/ Sodium Pyruvate (VWR, USA) supplemented with 2% (vol/vol) of decomplemented fetal bovine serum (FBS, Biowest, France) and 1% (vol/vol) Penicillin-Streptomycin (VWR, USA). The cells were grown at 37°C in a humidified 5% CO_2_ atmosphere. Cells were routinely passaged when they reached 100% confluence and were passaged until passage 15.

SARS-CoV-2 (strain BetaCov/Belgium/Sart-Tilman/2020/1, passage 5) (39). SARS-CoV-2 was isolated and propagated in Vero E6 cells. All the experiments involving SARS-CoV-2 were performed in a biosafety level 3 laboratory.

### Bacterial strains

A spontaneous mutant of a modified strain of *Bacillus licheniformis* MW3 comP::IS, ΔhsdR1, ΔhsdR2, tet, xylR-Pxyl::comK (called BBG 146-2), kindly provided by Max Béchet (Université de Lille), was used for the production of lichenysin. *Bacillus pumilus* QST 2808 (Agraquest, USA) was used to produce pumilacidin, and *Bacillus velezensis* GA1 was used (40) to produce iturin. Fusaricidin was produced using the *Paenibacillus polymyxa* pp56 strain (41).

### Lipopeptides

The lipopeptides selected for this study are summarized in Table 1.

**TABLE 1:** Lipopeptides selected for the screening

	Genus	Lipopeptide	Subfamily	Structure type	Fatty acid	Amino acid number	Charge	Amino acid sequence of the main representative	Purity (%)
Bacterial lipopeptides	*Bacillus* sp.	Surfactin	Surfactin	Cyclic	C12-C17	7	Anionic	L-Glu_1_-L-Leu_2_-D-Leu_3_-L-Val_4_-L-Asp_5_-D-Leu_6_-L-Leu_7_	90
			Lichenysin					L-Gln_1_-L-Leu_2_-D-Leu_3_-L-Val_4_-L-Asp_5_-D-Leu_6_-L-Ile_7_	>99
			Pumilacidin					L-Glu_1_-L-Leu_2_-D-Leu_3_-L-Leu_4_-L-Asp_5_-D-Leu_6_-L-Ile_7_	90
		Fengycin		Partially cyclic (D-Tyr_3_-L-Ile_10_)	C14-C18	10	Anionic	L-Glu_1_-D-Orn_2_-D-Tyr_3_-D-allo-Thr_4_-L-Glu_5_-D-Ala/Val_6_-L-Pro_7_-L-Gln_8_-L-Tyr_9_-L-Ile_10_	>99
		Iturin	Iturin A	Cyclic	C14-C17	7	Non-ionic	L-Asn_1_-D-Tyr_2_-D-Asn_3_-L-Gln_4_-L-Pro_5_-D-Asn_6_-L-Ser_7_	>99
			Mycosubtilin					L-Asn_1_-D-Tyr_2_-D-Asn_3_-L-Gln_4_-L-Pro_5_-D-Ser_6_-L-Asn_7_	>99
	*Paenibacillus* sp.	Fusaricidin		Cyclic	GHPD (C15)	6	Non-ionic	L-Thr_1_-D-Val_2_-L-Val/Tyr_3_-D-allo-Thr_4_-D-Asn/Gln_5_-D-Ala_6_	88
		Polymyxin	Polymyxin B	Partially cyclic (L-Dab_4_-L-Thr_10_)	C8-C9	10	Cationic	L-Dab_1_-L-Thr_2_-L-Dab_3_-L-Dab_4_-L-Dab_5_-D-Phe_6_-L-Leu_7_-L-Dab_8_-L-Dab_9_-L-Thr_10_	>99
			Colistin (polymyxin E)					L-Dab_1_-L-Thr_2_-L-Dab_3_-L-Dab_4_-L-Dab_5_-D-Leu_6_-L-Leu_7_-L-Dab_8_-L-Dab_9_-L-Thr_10_	>99
	*Streptomyces* sp.	Daptomycin		Partially cyclic (L-Thr_4_-L-Kyn_13_)	C10	13	Anionic	L-Trp_1_-L-Asn_2_-L-Asp_3_-L-Thr_4_-Gly_5_-L-Orn_6_-L-Asp_7_-D-Ala_8_-L-Asp_9_-Gly_10_-D-Ser_11_-3-MeGlu_12_-L-Kyn_13_	>95
	*Pseudomonas* sp.	Pseudofactin		Partially cyclic (Thr_3_-Leu/Val_8_)	C16	8	Non-ionic	Gly_1_-Ser_2_-Thr_3_-Leu_4_-Leu_5_-Ser_6_-Leu_7_-Leu/Val_8_^*a*^	91.2
		Viscosin	White line-inducing principle	Partially cyclic (D-allo-Thr_3_-L-Ile_9_)	C10-C12	9	Anionic	L-Leu_1_-D-Glu_2_-D-allo-Thr_3_-D-Val_4_-L-Leu_5_-D-Ser_6_-L-Leu_7_-D-Ser_8_-L-Ile_9_	99
		Orfamide	Orfamide B	Partially cyclic (D-allo-Thr_3_- L-Val_10_)	C12/C14	10	Anionic	L-Leu_1_-D-Glu_2_-D-allo-Thr_3_-D-Ile_4_-L-Leu_5_-D-Ser_6_-L-Leu_7_-L-Leu_8_-D-Ser_9_-L-Val_10_	>85
Fungal lipopeptides	*Aspergillus* sp.	Echinocandin	Caspofungin	Cyclic	C15, C16, C18	6	Cationic	(2-aminoethyl)amino)-N^2^-4-OH-L-Orn_1_-L-Thr_2_-trans-4-OH-L-Pro_3_-(S)−4-OH-4-(p-OH-ph)-L-Thr_4_- *threo*-3-OH-L-Orn_5_-trans-3-OH-L-Pro_6_	>98
	*Fusarium* sp.	Apicidin		Cyclic	C10	3	Non-ionic	Trp_1_-Ile_2_-Pip_3_	>95

^*a*^ L or D enantiomers have not been determined for pseudofactin.

Daptomycin, caspofungin (acetate), and orfamide B were purchased from Sanbio (the Netherlands), polymyxin B sulfate and colistin were obtained from Merck (Germany), apicidin and WLIP were obtained from Santa Cruz Biochemicals (USA), fengycin and mycosubtilin were obtained from Lipofabrik (France), and surfactin was obtained from Kaneka (Japan).

For the production of lichenysin, pumilacidin, and iturin, the strains were grown overnight (ON) in lysogeny broth (LB) (10 g/L NaCl (Merck), 5 g/L yeast extract (Thermo Fisher Scientific, USA), and 10 g/L tryptone (MP Biomedicals, USA)), at 37°C and 160 rpm. The ON cultures were used to inoculate Landy medium (42) at an optical density at 600 nm (OD600) of 0.1. For the production of lichenysin, Landy medium was modified to replace glucose with 10 g/L xylose and 5 g/L sucrose. *B. licheniformis* BBG 146-2 was incubated at 30°C and 160 rpm for 72 h; *B. pumilus* QST 2808 and *B. velezensis* GA1 were incubated at 37°C and 160 rpm for 48 h.

For the purification of lichenysin, pumilacidin, and iturin, the cells were harvested by centrifugation at 25,000 × *g* for 30 min at 4°C. The supernatant was acidified at pH 2 using H_2_SO_4_ and incubated ON at 4°C. The acidified supernatant was centrifuged, and the pellet was kept, resolubilized in H_2_O, and brought to pH 8 using NaOH. Liquid-liquid extraction (LLE) was performed with the same volume of a 7:3 (vol:vol) solvent mixture of ethyl acetate and butanol. The solvent phase was collected and dried using a SpeedDry RVC 2-25 CD Plus (Martin Christ, Germany) until it reached a 5 mL volume. The lipopeptide-containing fractions were further purified by preparative high-performance liquid chromatography (HPLC) (PuriFlash PF4250-250; Interchim, France). For the purification of lichenysin, a gradient of acetonitrile (ACN) in water acidified with 0.1% (vol/vol) trifluoroacetic acid (TFA) was used as follows: 10 min at 70% ACN, 10 to 70 min at 90% ACN, and 70 to 80 min at 100% ACN. For the purification of pumilacidin, the gradient used was 15 min at 60% ACN, 15 to 65 min at 85% ACN, and 65 to 85 min at 100% ACN. For the purification of iturin, the following gradient was used: 15 min at 20% ACN, 15 to 35 min at 30% ACN, 35 to 55 min at 50% ACN, 55 to 95 min at 80% ACN, and 95 to 105 min at 100% ACN.

For the production of fusaricidin, *P. polymyxa* pp56 was grown ON in LB at 37°C and 160 rpm. The ON culture was used to inoculate Katznelson & Lochhead (KL) media (43) at an OD600 of 0.1, which was subsequently incubated at 30°C and 160 rpm for 72 h.

For the purification of fusaricidin, LLE was performed directly on the cell supernatant with the same volume of solvent mixture as that described above and following the same steps. Fusaricidin was purified by preparative HPLC using the following gradient: 20 min at 30% ACN, 20 to 70 min at 30% to 80% ACN, 70 to 90 min at 80% ACN, and 90 to 110 min at 100% ACN.

The purity of the collected fractions was analyzed by ultra-performance liquid chromatography–mass spectrometry (UPLC-MS) as described below. Fractions containing only pure lichenysin, pumilacidin, iturin, or fusaricidin were pooled and concentrated using a SpeedDry RVC 2-25 CD Plus before being freeze-dried using an Alpha 3-4 LSCbasic (Martin Christ).

Lipopeptide stocks were dissolved in dimethyl sulfoxide (DMSO; Merck, Germany) at a concentration of 20 mg/mL and stored at −20°C.

### Lipopeptide detection, quantification, and structural confirmation by quadrupole time-of-flight - mass spectrometry (QTOF-MS)

To detect the lipopeptides and confirm their mass, UPLC-MS analyses were performed using an Acquity UPLC H-Class sample manager with a quaternary solvent manager and an SQ detector (Waters, USA). A 10 µL sample was injected on a Waters Acquity BEH C18 1.7 µm column (2.1 × 50 mm) with a C18 1.7 µm precolumn. The temperature was set at 40°C, and the flow rate was 0.6 mL/min. A gradient of ACN in water acidified with 0.1% (vol/vol) TFA was used as follows: from 30% ACN to 95% ACN in 2.4 min, maintained at 95% ACN until 5.1 min, from 95% ACN to 30% ACN in 0.1 min and maintained at 30% ACN until the end of the run (7 min). For detection, an electrospray in positive ion mode was used with the following parameters: source temperature at 130°C, desolvation temperature at 400°C, nitrogen flow at 1,000 L/h, and cone voltage at 120 V. Data acquisition and processing were performed using MassLynx, version 4.1.

For quantification, a Nexera UPLC-DAD (Shimadzu, Japan) was used with the same column, mobile phase, and run parameters as UPLC-MS. The DAD detector (diode array detector) was set to an analysis spectrum ranging from 190 to 800 nm. Quantification was performed by comparing the area of the lipopeptide analyzed with a calibration curve of an external surfactin standard (Kaneka) or iturin standard (Lipofabrik).

Structural characterization of the lipopeptides was performed by an liquid chromatography and electrospray ionization quadrupole time-of-flight mass spectrometry (LC-ESI-QTOF-MS) (Agilent 1290 Infinity II, USA) coupled with a mass detector (Jet Stream ESI-Q-TOF 6530) in positive mode with the following source parameters: capillary voltage of 3.5 kV, nebulizer pressure of 35 psi, drying gas of 8 L/min, gas temperature of 300°C, flow rate of sheath gas of 11 L/min, sheath gas temperature of 350°C, fragmentor voltage of 175 V, skimmer voltage of 65 V, and octopole radiofrequency of 750 V. Accurate mass spectra were recorded in the m/z range of 100 to 1,700 (acquisition rate 3 spectra/s). For optimal separation of the different homologs, a C18 Acquity UPLC BEH column (2.1 mm; 50 mm; 1.7 µm; Waters) was used at a flow rate of 0.6 mL/min and a temperature of 40°C (injection volume: 10 µL). A gradient of acidified water (0.1% formic acid) (solvent A) and acidified acetonitrile (0.1% formic acid) (solvent B) was chosen as the mobile phase starting at 2% B for 1 min before increasing to 80% B in 6.9 min. Then, solvent B was kept at 100% for 3 min before returning to the initial ratio. The main lipopeptide molecular ions were selected as precursors and further fragmented. Masshunter 10.0 software (Agilent) was used to process the data.

### RNA extraction and reverse transcription-quantitative polymerase chain reaction (RT-qPCR)

Here, RT-qPCR has been chosen over 50% tissue culture infectious dose (TCID50) and plaque assays for the detection and quantification of SARS-CoV-2 as it offers many advantages. It is highly sensitive and specific, capable of detecting low quantities of viral RNA. In addition, RT-qPCR provides rapid results, and allows precise quantification of viral load. Furthermore, some of the steps can be automated, and RT-qPCR is a high-throughput technique, making it the ideal technique for the screening of the 15 lipopeptides we have tested. Conversely, TCID50 and plaque assays are less sensitive, time-consuming, and low throughput, thus making RT-qPCR our clear choice for this study.

Total DNA and RNA were extracted from the samples using TANBead OptiPure Viral Auto plates (TANBead, Taiwan) in a Maelstrom 9600 (TANBead) following the manufacturer’s instructions. Single-step reverse transcription and qPCR were performed using a Luna Universal Probe One-Step RT-qPCR Kit (New England BioLabs, USA) according to the manufacturer’s instructions. RT-qPCR was used to amplify the N2 gene of SARS-CoV-2 via the use of 1.5 µL of a mixture of 10 µM SARS-CoV-2 N2 forward primer (5′-TTACAAACATTGGCCGCAAA-3′), the SARS-CoV-2 N2 reverse primer (5′-GCGCGACATTCCGAAGAA-3′), and the SARS-CoV-2 N2 probe (5′-ACAATTTGCCCCCAGCGCTTCAG-3′) (44). RT-qPCR was performed in a StepOne Real-Time PCR System (Thermo Fisher Scientific) or in a QuantStudio 1 Real-Time PCR System (Thermo Fisher Scientific) according to the manufacturer’s instructions with the following setup: 55°C for 10 min, 95°C for 1 min (hold stage), 95°C for 3 s, and 55°C for 30 s (cycle stage). The number of amplifying cycles was set at 45, and the results were considered positive if the Ct was less than 45. Amplification of β-actin mRNA was used as an internal control for RT-qPCR as described by Pirokowski et al. (45), using the same cycling conditions as mentioned above. The calculation of viral RNA copies was performed by absolute quantification based on a standard curve of synthetic RNA at known concentrations.

### Cytotoxicity

The cytotoxic activity of the lipopeptides was assessed using a CyQUANT XTT Cell Viability Assay (Thermo Fisher Scientific) according to the manufacturer’s instructions. Briefly, Vero E6 cells were seeded at a density of 20,000 cells per well in 96-well plates (96-well, Nunclon Delta-Treated, Thermo Fisher Scientific) and incubated with a range of lipopeptide concentrations. The cells were then incubated for 72 h at 37°C. The kit was then used following the manufacturer’s instructions, and the plates were read at 450 nm and 660 nm using a Multiskan GO Microplate Spectrophotometer (Thermo Fisher Scientific). The percentage of viable cells was calculated as (A_T450_ − A_T660_ − A_B450_) / (A_C450_ − A_C660_) × 100%, where A_T450_, A_T660_, A_B450_, A_C450_, and A_C660_ represent the absorbance of the tested well at 450 nm, of the tested well at 660 nm, of the blank (only medium) at 450 nm, of the control cells at 450 nm, and of the control cells at 660 nm, respectively. One hundred percent viability corresponds to the viability of uninfected cells. DMSO concentrations ranging from 0.5% (vol/vol) to 0.001% (vol/vol) were used as a negative control for the assay. Every sample was tested in triplicate.

### Replication

The effect of lipopeptides on the replication of SARS-CoV-2 was assessed with slight modifications to the cytotoxicity protocol. Vero E6 cells were seeded at a density of 20,000 cells per well in 96-well plates, directly incubated with a range of lipopeptide concentrations and infected at a multiplicity of infection (MOI) of 0.01. Every sample was tested in triplicate. Positive neutralizing serum with a seroneutralization titer of 1/1,280 was diluted five times and used as a positive control. DMSO at the corresponding concentrations as used to dissolve the lipopeptides was used as a negative control. The plates were then incubated for 72 h at 37°C, after which cell supernatant samples were taken every 24 h. Each sample was inactivated for 30 min at 70°C before absolute quantification of the viral RNA by RT-qPCR based on a calibration curve generated from the synthetic RNA.

### Direct antiviral assay by viral titration

To determine the potential inactivation of SARS-CoV-2 by lipopeptides, titration of residual infectious virions was performed. Lipopeptides and undiluted viral stock of SARS-CoV-2 were mixed and incubated for 1 h at 37°C. Subsequently, 10-fold serial dilutions of the viral stock or mixture were performed in 96-well plates before the addition of Vero E6 cells at a density of 20,000 cells per well. Positive neutralizing serum with a seroneutralization titer of 1/1,280 was diluted five times and used as a positive control. DMSO at the corresponding concentrations as used to dissolve the lipopeptides was used as a negative control. The plates were then incubated for 5 days at 37°C, after which the CPE for each well was determined using a Primovert optical microscope (Zeiss, Germany). The TCID_50_ was then calculated using the Reed-Muench method and is expressed as the log (TCID_50_) (46).

### Binding inhibition assay

**Fig 1:**
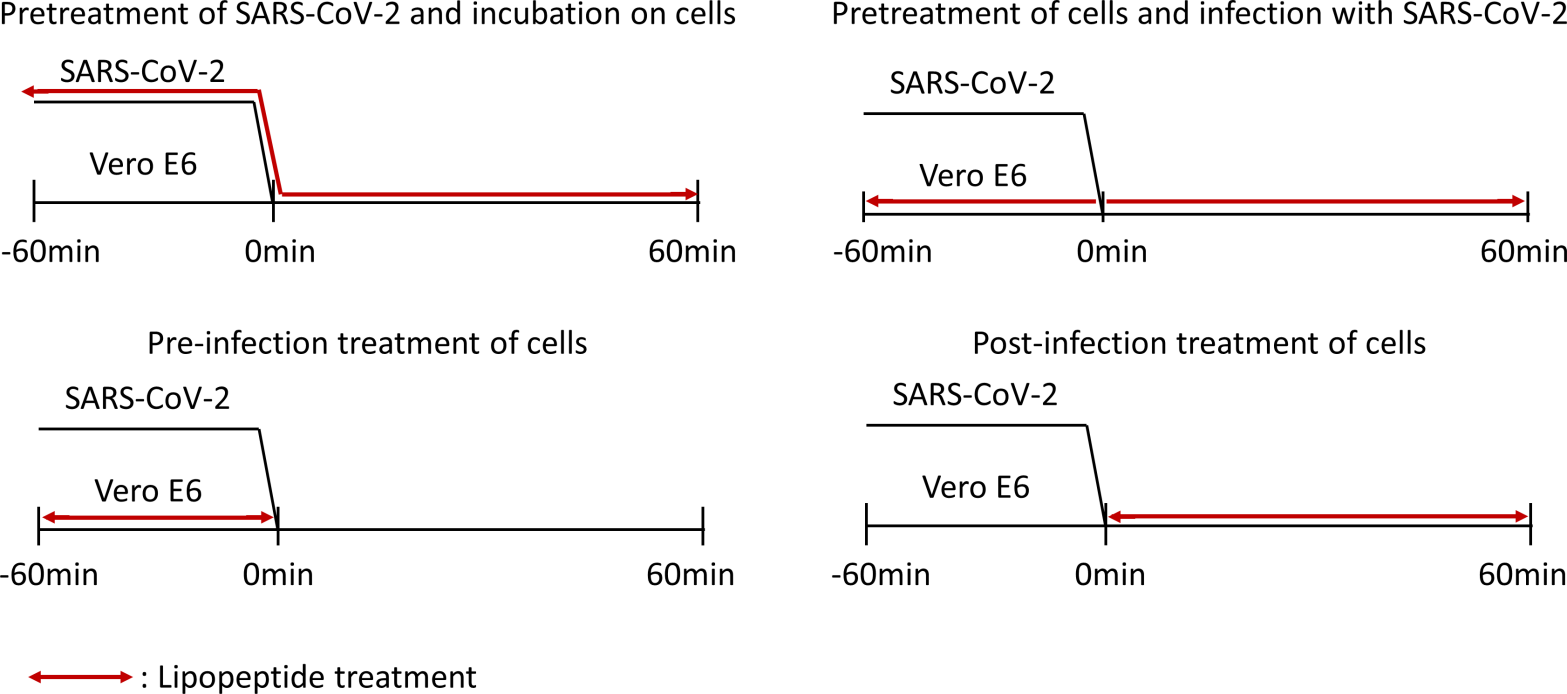
Conditions of the binding inhibition experiments. Four different binding inhibition assays were conducted to investigate the potential inhibitory effect of lipopeptides on the binding of SARS-CoV-2 to Vero E6 cells. Infected cells treated with DMSO were used as a negative control for the assays.

For the binding inhibition assays, four different conditions (Fig. 1) were tested to assess the potential of lipopeptides to inhibit the attachment of SARS-CoV-2 to cells in different settings. For these assays, Vero E6 cells were seeded at a density of 20,000 cells per well in 96-well plates in DMEM + 10% (vol/vol) FBS and incubated ON to allow the cells to adhere to the surface of the wells. In these assays, SARS-CoV-2 was used at an MOI of 10, and every sample was tested in triplicate. Some steps were performed at 4°C to rigidify the cellular and viral membranes, allowing the virus to bind to the cells but not fuse with the virus, as detailed below.

#### Pre-treatment of SARS-CoV-2 and incubation on cells

Lipopeptides were incubated with SARS-CoV-2 for 1 h at 37°C with gentle agitation. Positive neutralizing serum with a seroneutralization titer of 1/1,280 was diluted five times and used as a positive control. DMSO at the corresponding concentrations as used to dissolve the lipopeptides was used as a negative control. The viral mixture and the cells were pre-chilled for 15 min on ice. The cell supernatant was discarded, and the viral mixture was added to the cells. The inoculated cells were then incubated for 1 h on ice with gentle agitation. The inoculums were discarded, and the cells were gently washed three times with ice-cold Dulbecco’s phosphate buffered saline (DPBS; VWR, USA) before the addition of 200 µL of DPBS to each well. The plates were inactivated for 30 min at 70°C before quantification of the viral RNA by RT-qPCR.

#### Pre-treatment of cells and infection with SARS-CoV-2

The cell supernatant was discarded, and the lipopeptides were added to the cells, which were subsequently incubated for 1 h at 37°C with gentle agitation. Positive neutralizing serum with a seroneutralization titer of 1/1,280 was diluted five times and used as a positive control. DMSO at the corresponding concentrations as used to dissolve the lipopeptides was used as a negative control. The viral stock (MOI of 10) and the cells were pre-chilled for 15 min on ice. The cells were then infected with the cold virus stock and incubated for 1 h on ice with gentle agitation. The inoculums were discarded, and the cells were gently washed three times with ice-cold DPBS before the addition of 200 µL/well of DPBS. The samples were inactivated for 30 min at 70°C before quantification of the viral RNA by RT-qPCR.

#### Pre-infection treatment of cells

The cell supernatant was discarded, and the lipopeptides were added to the cells, which were subsequently incubated for 1 h at 37°C with gentle agitation. DMSO at the corresponding concentrations as used to dissolve the lipopeptides was used as a negative control. The viral stock (MOI of 10) and the cells were pre-chilled for 15 min on ice. The lipopeptides were removed, and the cells were gently washed three times with ice-cold DPBS. The viral stock was then added to the cells, which were subsequently incubated on ice for 1 h with gentle agitation. The supernatants were discarded, and the cells were gently washed three times with ice-cold DPBS before the addition of 200 µL/well of DPBS. The samples were inactivated for 30 min at 70°C before quantification of the viral RNA by RT-qPCR.

#### Post-infection treatment of cells

The cells, lipopeptides, and viral stock (MOI of 10) were pre-chilled for 15 min on ice. DMSO at the corresponding concentrations as used to dissolve the lipopeptides was used as a negative control. The cell supernatant was removed, and the lipopeptides and viral stock were added simultaneously to the cells, which were subsequently incubated for 1 h on ice with gentle agitation. The inoculum was discarded, and the cells were gently washed three times with ice-cold DPBS before the addition of 200 µL/well of DPBS. The samples were inactivated for 30 min at 70°C before quantification of the viral RNA by RT-qPCR.

### Fusion inhibition assay

To determine the impact of lipopeptides on the fusion step, intracellular viral RNA was quantified after infection of cells with SARS-CoV-2 but before the budding step. Vero E6 cells were seeded at a density of 20,000 cells per well in 96-well plates in DMEM + 10% (vol/vol) FBS and incubated ON. The cells were pre-chilled for 15 min on ice. The cells were infected with SARS-CoV-2 (MOI of 10) in DMEM + 2% FBS and incubated for 2 h on ice with gentle agitation. The inoculum was removed, and the cells were gently washed three times with ice-cold DPBS. The positive control was inactivated to determine the basal viral RNA concentration after binding of the virus. The lipopeptides were added to room temperature DMEM + 2% (vol/vol) FBS and incubated at 37°C for 7 h. DMSO at the corresponding concentrations as used to dissolve the lipopeptides was used as a negative control. The cells were rinsed with DPBS, and the wells were dried. The samples were inactivated for 30 min at 70°C before quantification of the viral RNA by RT-qPCR. Every sample was tested in triplicate.

### Budding inhibition assay

For the budding inhibition assay, Vero E6 cells were seeded at a density of 20,000 cells per well in 96-well plates in DMEM + 10% (vol/vol) FBS and incubated ON. The cell supernatant was discarded, and the cells were infected with SARS-CoV-2 at an MOI of 10 in DMEM + 2% FBS and incubated at 37°C for 1 h with gentle agitation. The inoculum was removed, and the cells were gently washed three times with DPBS to remove unbound virions. Fresh medium was added to the cells, and the plates were incubated for 5 h at 37°C. The supernatant was discarded, and lipopeptides were added to the cells. A sample of cell supernatant was taken every hour for 4 h. After infection, the control was kept at 4°C to inhibit the release of virions, and act as a positive control of the experiment. DMSO at the corresponding concentrations as used to dissolve the lipopeptides was used as a negative control. The samples were inactivated for 30 min at 70°C before quantification of the viral RNA by RT-qPCR. Every sample was tested in triplicate.

### Statistical analysis

The half maximal inhibitory concentration (IC_50_) was calculated, and one-way analysis of variance (ANOVA) followed by Dunnett’s multiple comparisons test was performed using GraphPad Prism 9.1.0.221 for Windows (GraphPad Software, San Diego, California, USA).

The correlations between the different structural variables and antiviral activity were determined by partial least squares regression using XLSTAT 2023.1.5 (1409) (ADDINSOFT, Paris, France).

## RESULTS

### Selection of the lipopeptide

A literature review was performed to explore the wide diversity of lipopeptides and how this structural variety leads to various biological effects. This review aimed to select a subset of diverse lipopeptides for studying their structure-activity relationships.

For a compound to be classified as a lipopeptide, it had to meet two criteria: first, the fatty acid chain should be longer than the longest amino acid carbon chain (leucine with six carbons). Second, the peptidic chain making up the lipopeptide should consist of at least two amino acids forming a peptide bond. Because lipopeptides are biosynthesized by the NRPS, they exhibit extensive diversity, with more than 120 lipopeptide families meeting our criteria (Table S1).

To cover the broad structural diversity of lipopeptides, 15 different lipopeptides with distinct structural shapes (cyclic, partially cyclic, or linear), FA chain lengths (from C8 to C18), amino acid numbers (from 3 to 13), and charges (anionic, cationic, or non-ionic) were selected for testing their cytotoxicity and antiviral activity against SARS-CoV-2 (Table 1). Interestingly, among the selected lipopeptides, surfactin, fengycin, and viscosin have demonstrated an antiviral activity.

### Screening of the lipopeptides for their cytotoxicity

**Fig 2:**
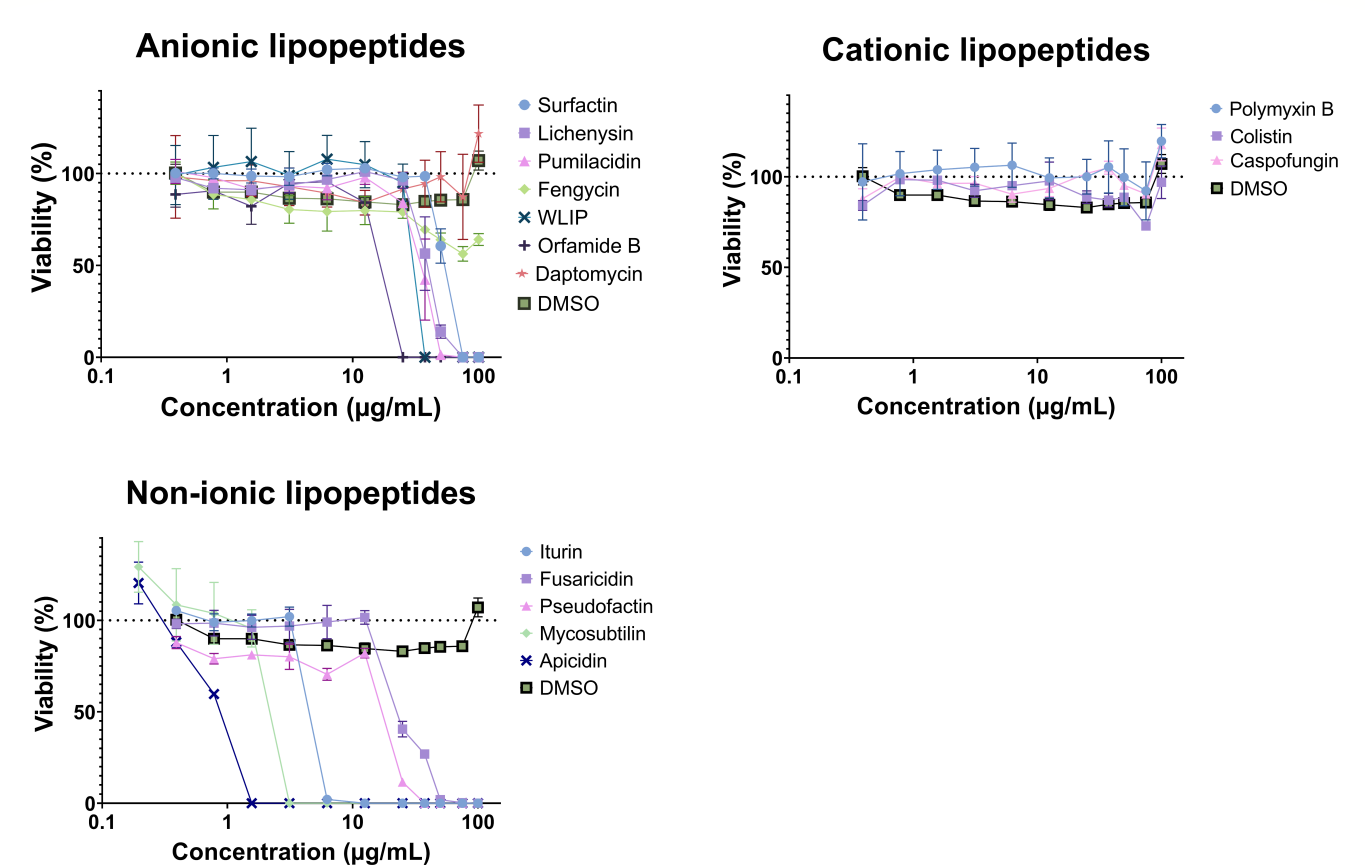
Cytotoxicity of the lipopeptides as a function of their concentration. The cytotoxic activity of the 15 lipopeptides was assessed by incubating the lipopeptides for 72 h with Vero E6 cells and measuring the activity using XTT reagent. One hundred percent viability corresponds to the viability of uninfected cells. DMSO concentrations ranging from 0.5% (vol/vol) to 0.001% (vol/vol) were used as a negative control for the assay. Each point corresponds to the mean of a triplicate with the standard deviation.

These lipopeptides were screened for their cytotoxicity on Vero E6 cells at concentrations ranging from 0.2 µg/mL to 100 µg/mL. The cytotoxicity of the lipopeptides was investigated by incubating the cells with different concentrations of lipopeptides and determining their viability (Fig. 2). DMSO from 0.5% (vol/vol) to 0.001% (vol/vol) was used as a negative control. Globally, non-ionic lipopeptides decrease cell viability to 0% at lower concentrations (between 1.6 and 50 µg/mL) than charged lipopeptides. Most anionic lipopeptides are cytotoxic at concentrations ranging between 12.5 and 100 µg/mL, and cationic lipopeptides are non-cytotoxic at the concentrations tested. Among non-ionic lipopeptides, apicidin is the most cytotoxic. Among the anionic lipopeptides tested, fengycin had the lowest impact on cell viability, which was still 64.1% after treatment with 100 µg/mL fengycin. In the section Structural traits impacting cytotoxicity and antiviral activity, higher concentrations of fengycin and caspofungin were tested to determine their cytotoxicity at concentrations higher than 100 µg/mL.

The half maximal inhibitory concentration was determined for each lipopeptide (Table 2). Of the lipopeptides tested, fengycin exhibited the highest IC_50_ (295.3 µg/mL), while apicidin had the lowest IC_50_ (0.7 µg/mL). Among the lipopeptides from *Bacillus* sp., the iturin family (iturin and mycosubtilin) had the lowest IC_50_ values (4.9 and 1.8 µg/mL, respectively). Among the lipopeptides from *Pseudomonas* sp., WLIP had the highest IC_50_ (29.9 µg/mL).

**TABLE 2:** Cytotoxicity and antiviral activity of lipopeptides—characteristic parameters^*a*^

Lipopeptide	IC_50_ (µg/mL)	EC_50_ (µg/mL)	Maximal RNA reduction at 100 µg/mL
Surfactin	51.5 (1.4)	50.0 (6.5)	6.3-log
Lichenysin	28.1 (1.4)	25.9 (3.2)	6.3-log
Pumilacidin	36.0 (3.5)	47.8 (0.4)	6.3-log
Fengycin	295.3 (28.3)^*b*^	71.7 (20.7)	2.5-log
WLIP	29.9 (1.9)	18.4 (1.6)	6.3-log
Orfamide B	14.6 (0.4)	12.8 (4.1)	6.3-log
Daptomycin	>100.0	>100.0	0.3-log
Polymyxin B	>100.0	>100.0	0.3-log
Colistin	>100.0	>100.0	0.2-log
Caspofungin	128.9 (1.0)^*b*^	139.9 (17.2)	3.7-log
Iturin	4.9 (0.5)	18.2 (1.8)	3.9-log
Mycosubtilin	1.8 (0.1)	12.9 (2.0)	4.1-log
Fusaricidin	24.6 (1.1)	51.2 (13.6)	3.3-log
Pseudofactin	23.1 (0.3)	34.5 (3.5)	6.3-log
Apicidin	0.7 (0.1)	25.2 (1.3)	3.7-log

^*a*^ The IC_50_ and EC_50_ are expressed as the mean (standard deviation). IC_50_, half maximal inhibitory concentration; EC_50_, half maximal effective concentration.

^*b*^ IC_50_ determined by testing higher concentrations of these lipopeptides in a subsequent experiment, as their IC_50_ could not be determined at 100 µg/mL.

### Screening of the antiviral activity of the lipopeptides

**Fig 3:**
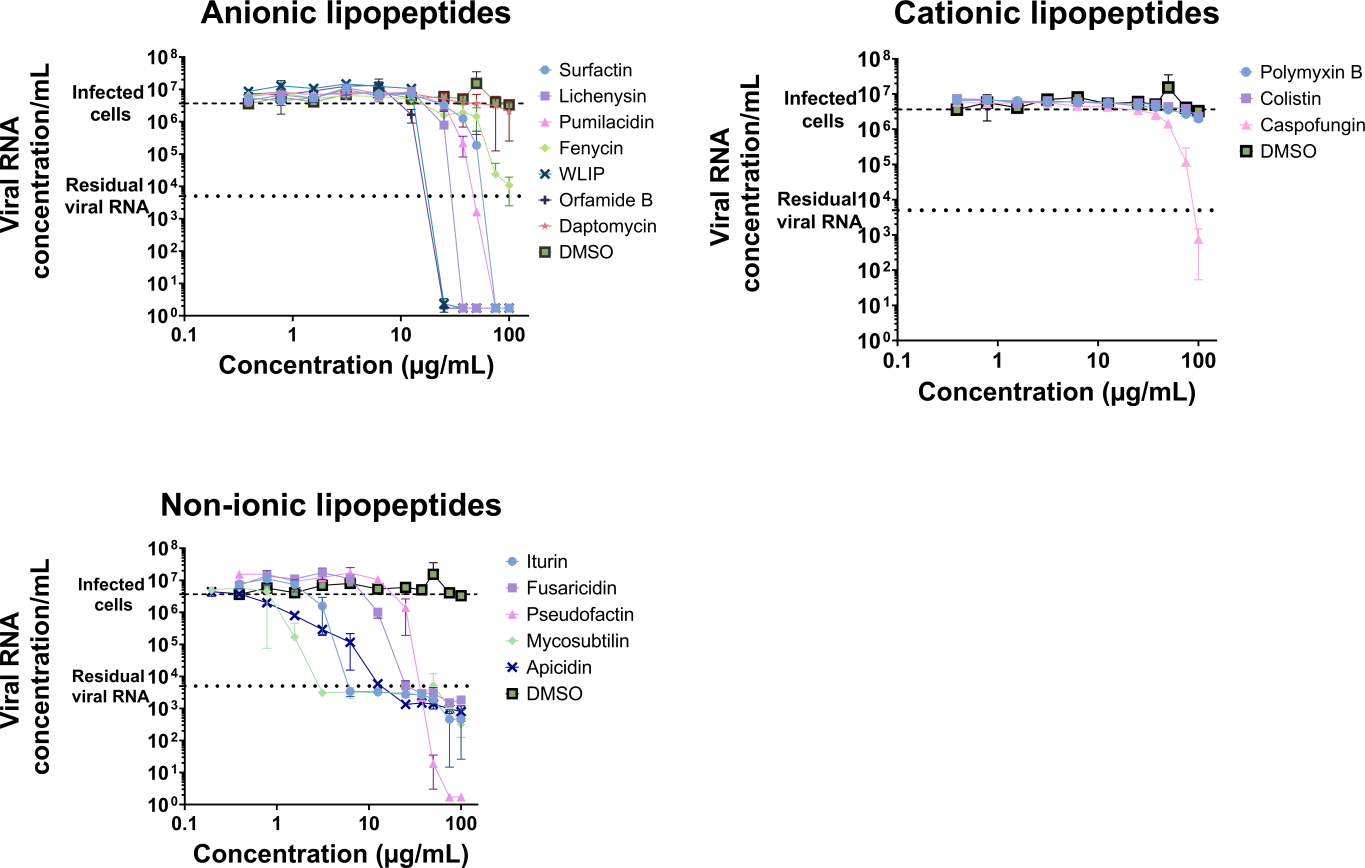
Antiviral activity of the lipopeptides as a function of their concentration. The antiviral activity of the 15 lipopeptides was assessed by incubating the lipopeptides with SARS-CoV-2 and Vero E6 cells for 72 h before quantifying the viral RNA concentration via RT-qPCR. The heavy dashed line “infected cells” corresponds to the RNA concentration per milliliter obtained with Vero cells infected with SARS-CoV-2 at a multiplicity of infection of 0.01 (no treatment), and the light dashed line “residual viral RNA” corresponds to SARS-CoV-2 at an MOI of 0.01 in medium without cells. DMSO was used as a negative control for the assay. Each point corresponds to the mean of a triplicate with the standard deviation.

The antiviral activity of the different lipopeptides was assessed by incubating different concentrations of lipopeptides with SARS-CoV-2-infected Vero cells and quantifying the concentration of viral RNA at 72 h post infection (pi) via RT-qPCR (Fig. 3). To evaluate the antiviral activity of the lipopeptides, two parameters were considered: the half maximal effective concentration (EC_50_) and the maximal RNA concentration reduction at 100 µg/mL compared to those in untreated infected cells (Table 2). Globally, anionic lipopeptides decrease the viral RNA concentration below the residual value at concentrations lower than those of the two other lipopeptide categories, and cationic lipopeptides exhibit the lowest antiviral activity.

Among the anionic lipopeptides tested, surfactin, lichenysin, pumilacidin, WLIP, and orfamide B were able to reduce the RNA concentration by 6.3-log at 100 µg/mL to undetected levels according to RT-qPCR. Fengycin reduced the RNA concentration by 2.5-log, but daptomycin had no effect. In the non-ionic lipopeptide category, only pseudofactin reduced the RNA concentration by 6.3-log, whereas the other lipopeptides reduced the RNA concentration by 3.3- to 4.1-log. Among the cationic lipopeptides tested, caspofungin has moderate antiviral activity (a 3.7-log reduction in RNA concentration), while the polymyxin family (polymyxin B and colistin) has no activity up to 100 µg/mL.

To select the best lipopeptides with high antiviral activity and the lowest cytotoxic activity, their IC_50_ and EC_50_ were compared. The EC_50_ was lower than the IC_50_, indicating antiviral activity at a concentration lower than the cytotoxic concentration for surfactin, lichenysin, WLIP, and orfamide B. In addition, fengycin and caspofungin reduced the RNA concentration at 100 µg/mL without any apparent cytotoxicity for caspofungin at 100 µg/mL and with a limited cytotoxicity for fengycin at 100 µg/mL (64.1% viability) (Fig. 2).

### Structural traits impacting cytotoxicity and antiviral activity

**Fig 4:**
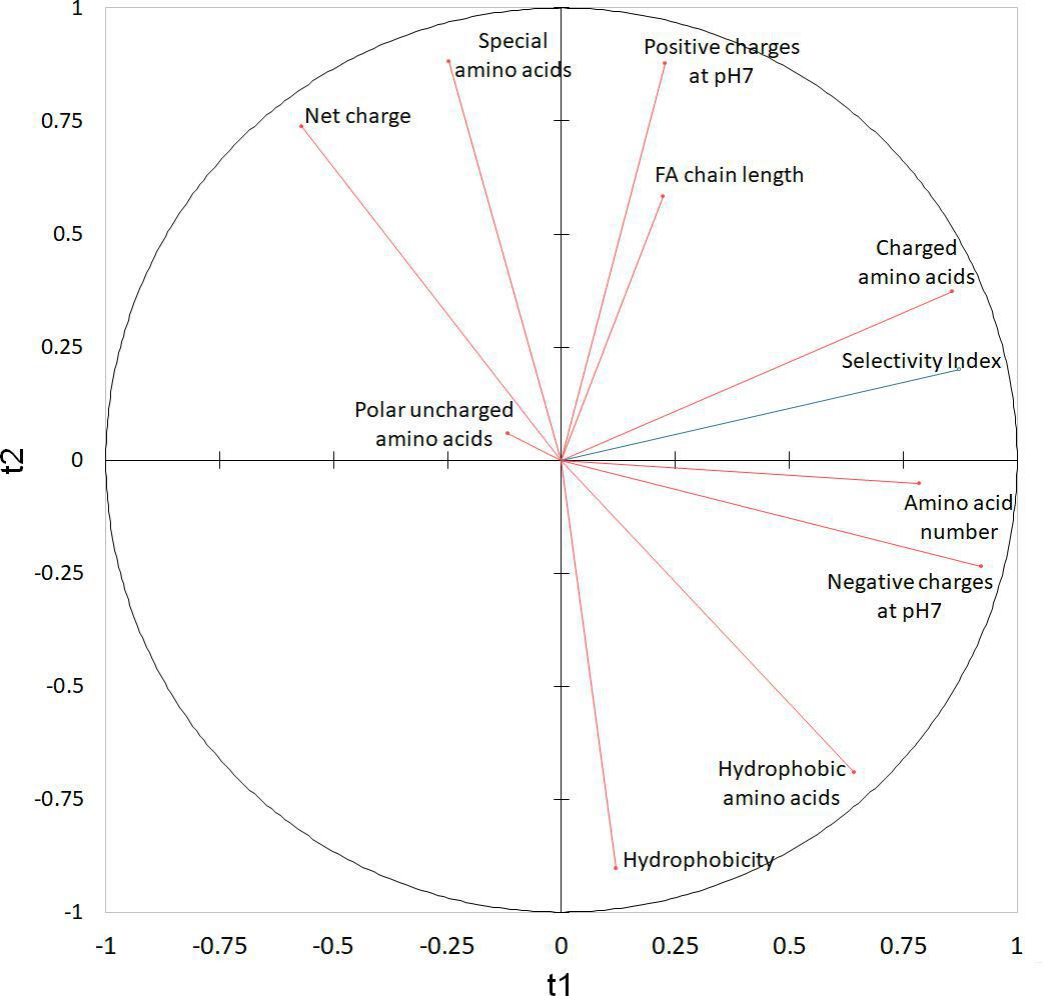
Correlation map with the two first components t1 (R^2^Y = 0.763; R^2^X = 0.313) and t2 (R^2^Y = 0.040; R^2^X = 0.392). The target variable (selectivity index) and the predictor variables are represented in blue and red, respectively.

A partial least square regression (PLSR) analysis (Fig. 4) was performed for a more in-depth investigation of the structure-function relationships. In this analysis, the target variable was the selectivity index corresponding to the ratio between the IC_50_ and EC_50_, and the predictor variables chosen to characterize the lipopeptide structure were the amino acid residue number, FA chain length, net charge, positive and negative charges at pH 7, hydrophobicity (determined using the grand average of hydropathy [GRAVY] index), number of charged, polar uncharged, special (cysteine, glycine, proline), and hydrophobic amino acids. PLSR analysis was performed on 9 out of the 15 lipopeptides, namely surfactin, fengycin, iturin, fusaricidin, pseudofactin, WLIP, orfamide B, caspofungin, and apicidin. Lichenysin, pumilacidin, and mycosubtilin were excluded to avoid bias toward a single lipopeptide family. Polymyxin B, colistin, and daptomycin were not analyzed due to incalculable selectivity indexes. PLSR identified the number of charged amino acids, the amino acid number, and the negative charges at pH 7 as the main variables positively correlated with the selectivity index. The FA chain length, positive charge at pH 7, and hydrophobic amino acids showed slight positive correlations, while the other structural variables demonstrated no correlation.

### Selection of promising lipopeptides with antiviral activity against SARS-CoV-2 (surfactin, WLIP, fengycin, and caspofungin)

**Fig 5:**
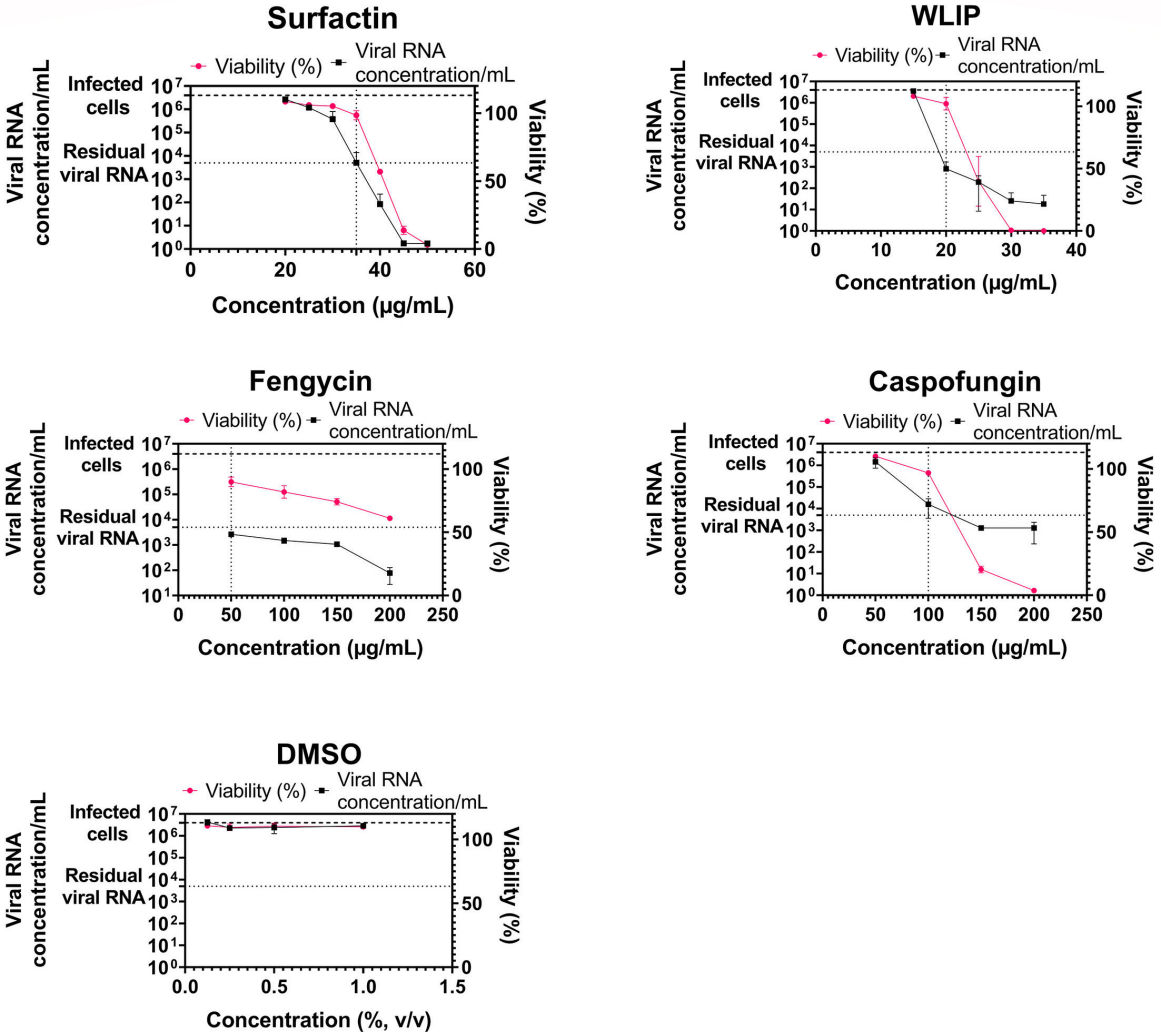
Range of concentrations tested in cytotoxicity and antiviral assays to select the best lipopeptide concentration for the subsequent assays. The concentrations tested ranged from 20 to 50 µg/mL and 15 to 35 µg/mL for surfactin and WLIP, respectively, and 50–200 µg/mL for fengycin and caspofungin. Cytotoxic activity was assessed by incubating the lipopeptides for 72 h with Vero E6 cells and measuring the activity using XTT reagent. One hundred percent viability corresponds to the viability of uninfected cells. Antiviral activity was assessed by incubating the lipopeptides with SARS-CoV-2 and Vero E6 cells for 72 h before quantifying the viral RNA concentration via RT-qPCR. The heavy dashed line “infected cells” corresponds to the RNA concentration per milliliter obtained with Vero cells infected with SARS-CoV-2 at an MOI of 0.01 (untreated), and the light dashed line “residual viral RNA” corresponds to SARS-CoV-2 at an MOI of 0.01 in medium without cells. DMSO was used as a negative control for both assays. Each point corresponds to the mean of a triplicate with the standard deviation.

Following the initial screening and PLSR analysis, four lipopeptides, namely surfactin, WLIP, fengycin, and caspofungin, were selected for a more in-depth investigation into the mechanism of their antiviral activity. In a first step, a broader range of concentrations was tested (Fig. 5) to identify the concentration at which viral RNA reduction occurs while maintaining a minimum of 80% viability considered non-toxic according to ISO 10993–5 (2009). For surfactin, a concentration of 35 µg/mL resulted in a 2.9-log reduction in viral RNA and a viability of 98.5%. WLIP at 20 µg/mL resulted in a 3.7-log decrease in viral RNA without affecting viability. Fengycin, at 50 µg/mL, maintained 89.9% viability and reduced viral RNA levels by 3.2-log. Caspofungin, at 100 µg/mL, yielded a viability of 96.9% and a 2.4-log reduction in viral RNA.

### Mode of action of the selected lipopeptides

To explore the mechanism of their antiviral activity, various assays targeting key elements of the virus infection process were conducted. The potential direct inhibitory effect of the lipopeptides on SARS-CoV-2 was also analyzed, as was their impact on virus replication. In addition, the steps involving the viral envelope and the host cell membrane, such as binding, fusion, and budding, were investigated.

#### Effect on viral replication

**Fig 6:**
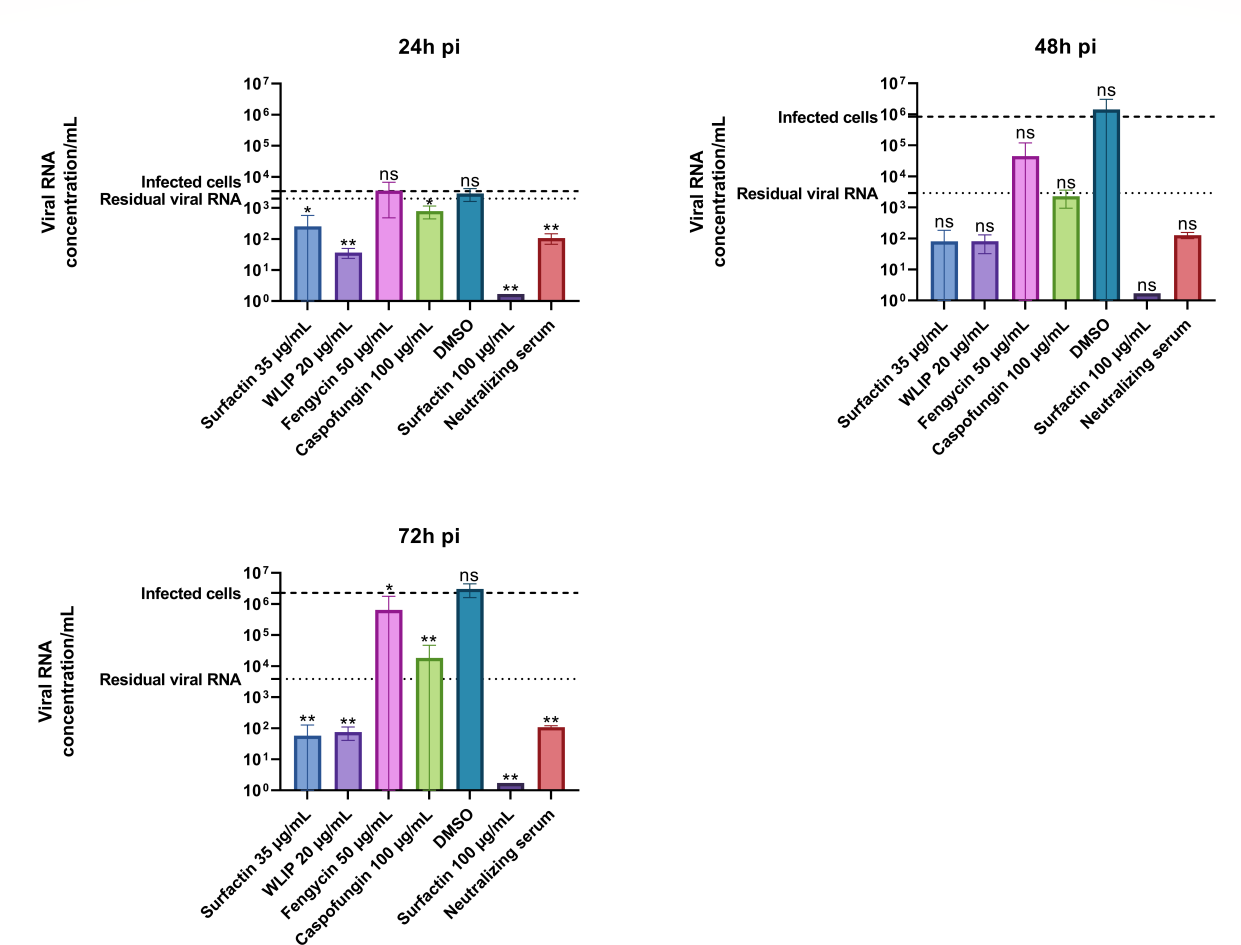
Effects of surfactin, WLIP, fengycin, and caspofungin on the replication of SARS-CoV-2 at 24 h, 48 h, and 72 h. The effect on the replication of SARS-CoV-2 was assessed by incubating the lipopeptides with SARS-CoV-2 and Vero E6 cells for 72 h and taking aliquots every 24 h before quantifying the viral RNA concentration via RT-qPCR. The heavy dashed line “infected cells” corresponds to the RNA concentration per milliliter obtained with Vero cells infected with SARS-CoV-2 at an MOI of 0.01 (untreated), and the light dashed line “residual viral RNA” corresponds to SARS-CoV-2 at an MOI of 0.01 in medium without cells. DMSO was used as a negative control for both assays. Each point corresponds to the mean of a triplicate with the standard deviation. Statistical significance (ns, *P* > 0.05; *, *P* ≤ 0.05; **, *P* ≤ 0.01; ***, *P* ≤ 0.001; ****, *P* ≤ 0.0001) was calculated for each condition versus “infected cells” by one-way ANOVA followed by Dunnett’s multiple comparisons test.

To evaluate the effect of lipopeptides on the replication of SARS-CoV-2, surfactin at 35 µg/mL, WLIP at 20 µg/mL, fengycin at 50 µg/mL, and caspofungin at 100 µg/mL were incubated with Vero E6 cells and SARS-CoV-2 for 72 h, after which the amount of viral RNA released into the supernatant was quantified at different timepoints (Fig. 6).

The viral RNA concentration in infected cells (dashed line, no treatment) greatly increased from 24 h (3.5 × 10^3^ RNA concentration per milliliter) to 72 h pi (2.3 × 10^6^ RNA concentration per milliliter), while the concentration of residual viral RNA (SARS-CoV-2 in medium without cells) remained at similar levels, between 2 × 10^3^ RNA concentration/mL and 3.8 × 10^3^ RNA concentration/mL, throughout the experiment. The concentration per milliliter of viral RNA obtained with DMSO, the negative control, at 24 h, 48 h, and 72 h pi was equal to that of the infected cells. Surfactin at 100 µg/mL was used as a positive control for reducing the viral RNA concentration, as we previously observed (Fig. 6) that at 45 µg/mL surfactin, the viability was close to 0%, and no viral RNA was detected. At every timepoint, surfactin at 100 µg/mL reduced the SARS-CoV-2 RNA concentration to non-detectable levels. Neutralizing serum was used as another positive control because it prevents the infection of cells by SARS-CoV-2 virions and thus blocks viral replication. The neutralizing serum was able to reduce the concentration of viral RNA by 1.5-log (to an RNA concentration per milliliter of 1 × 10^2^) at 24 h pi. This RNA concentration stayed at the same level at 48 h pi and 72 h pi while the viral RNA concentration in infected cells without treatment increased (corresponding to a 3.8-log and 4.3-log reduction, respectively). Compared to that of infected cells without treatment, surfactin at 35 µg/mL was able to reduce the concentration of viral RNA as soon as 24 h pi by 1.1-log (to an RNA concentration per milliliter of 2.5 × 10^2^), and the concentration of RNA decreased to reach 5.7 × 10^1^ RNA concentration per milliliter at 72 h pi (corresponding to a 4.6-log reduction). A similar trend was observed for WLIP at 20 µg/mL, with a reduction in the RNA concentration at 3.6 × 10^1^ RNA concentration per milliliter as soon as 24 h pi (2.0-log reduction), and a slight increase in the RNA concentration at 72 h pi resulted in a 4.5-log reduction (RNA concentration per milliliter of 7.5 × 10^1^ at 72 h pi). Fengycin at 50 µg/mL did not reduce the RNA concentration at 24 h pi. However, at 72 h pi, fengycin treatment reduced the RNA concentration by 0.6-log compared to the infected cells. Finally, caspofungin treatment at 100 µg/mL slightly reduced the RNA concentration at 24 h pi (to 7.9 × 10^2^ RNA concentration per milliliter), and the RNA concentration increased at 48 and 72 h pi to 1.8 × 10^4^ at 72 h pi, reducing the RNA concentration by 2.1-log compared to infected cells without treatment.

In these assays, surfactin (at 35 µg/mL) and WLIP (at 20 µg/mL) were able to reduce the viral RNA concentration to levels below the residual viral RNA at every timepoint and to levels similar to those of the neutralizing serum. Caspofungin was also able to reduce the RNA concentration compared to that in infected cells to levels similar to the residual viral RNA concentration at every timepoint. Finally, the effect of fengycin on the RNA concentration was inferior to that of the other three lipopeptides and could only be observed at 72 h pi.

#### Direct effect on SARS-CoV-2

**Fig 7:**
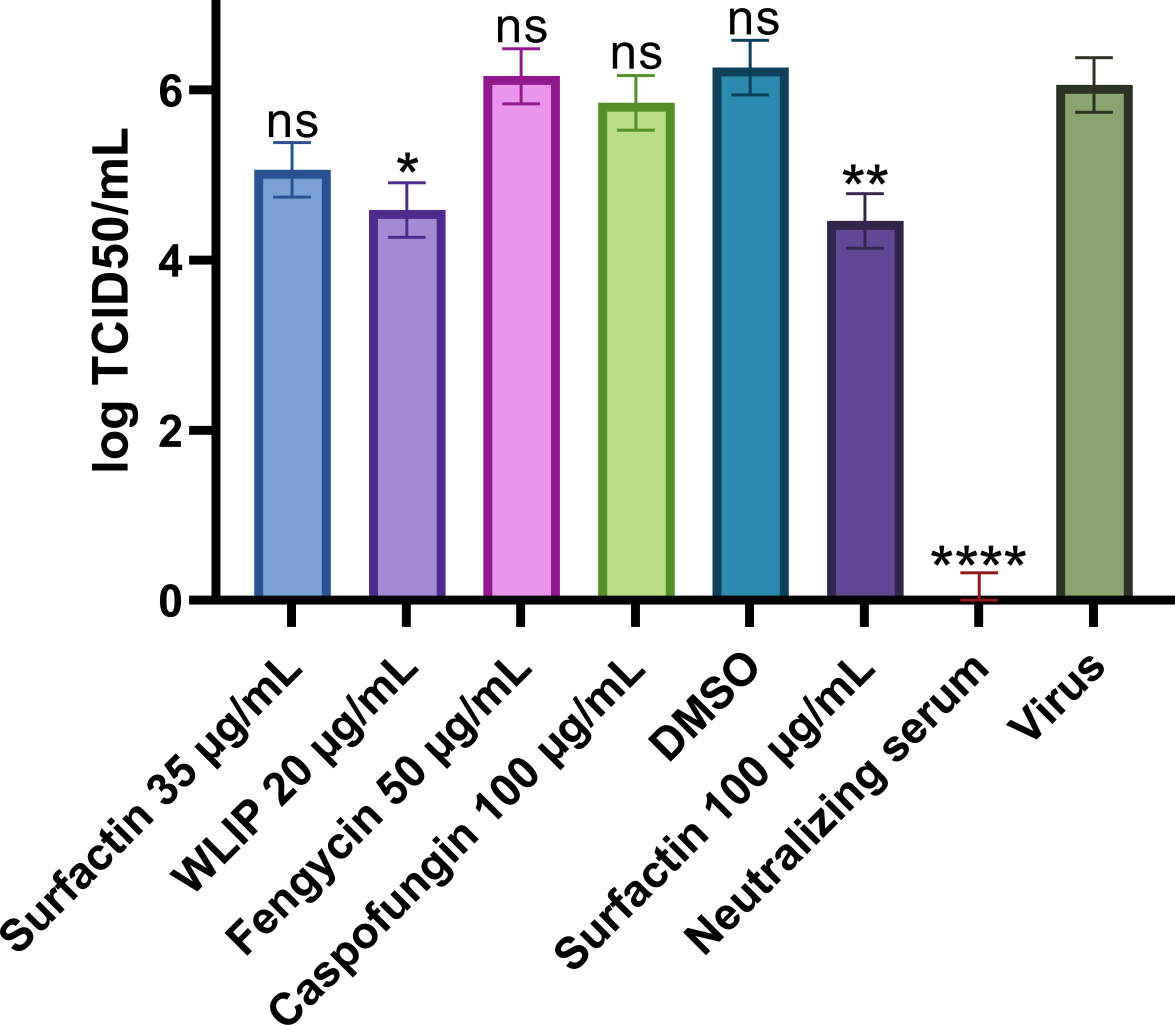
Direct effect of surfactin, WLIP, fengycin, and caspofungin on SARS-CoV-2. The lipopeptides were incubated with SARS-CoV-2 for 1 h before the residual virus was titrated, and the TCID_50_ per milliliter was determined by the Reed-Muench method. Each titer was determined using eight wells per dilution and is shown as the mean with the standard deviation. Statistical significance (ns, *P* > 0.05; *, *P* ≤ 0.05; **, *P* ≤ 0.01; ***, *P* ≤ 0.001; ****, *P* ≤ 0.0001) was calculated for each condition versus “virus,” corresponding to the titer of untreated virus, by use of a one-way ANOVA followed by Dunnett’s multiple comparisons test.

To study the potential direct effect of lipopeptides on SARS-CoV-2 in the absence of host cells, the four representative lipopeptides were incubated with SARS-CoV-2 for 1 h, after which the residual virus was titrated (Fig. 7). The neutralizing serum completely inactivated the virus after an hour of incubation, and no CPE was observed at the lowest concentration used for the titration. Surfactin at 100 µg/mL, used as a positive control in the replication assay, showed a significant reduction in the viral titer of 1.6-log compared to the virus alone. Of the four lipopeptides tested, only WLIP at 20 µg/mL had a significant reduction in viral titer (a 1.5-log reduction), while the other three lipopeptides did not significantly reduce the viral titer at the selected concentration.

#### Binding inhibition

Several experiments were performed at our laboratory with relevant controls to determine the timing of the binding of SARS-CoV-2 in Vero E6 cells (data not shown).

**Fig 8:**
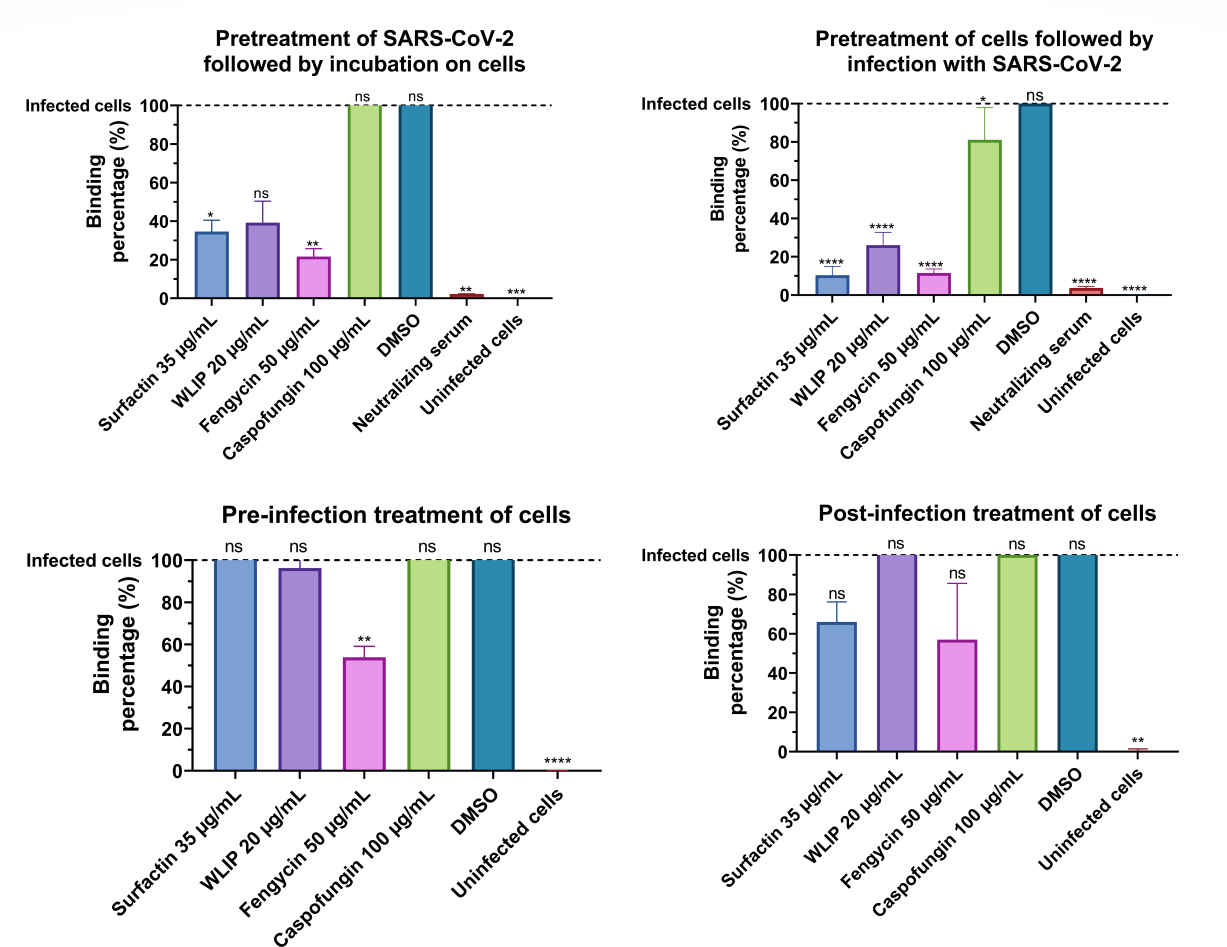
Binding inhibition assay of SARS-CoV-2 by surfactin, WLIP, fengycin, and caspofungin. The virus was pre-treated with lipopeptides before assessing its binding to Vero cells (pre-treatment of virus), the cells were pre-treated with lipopeptides before assessing the binding of the virus to pre-treated cells (pre-treatment of cells), the cells were pre-treated with lipopeptides before washing away the lipopeptides and assessing the binding of the virus to the cells (pre-infection), and lipopeptides were added simultaneously with the virus to the cells and assessing the binding of the virus (post-infection). The values were normalized against those for infected cells (dashed line, no treatment) and are plotted as the means of triplicate samples with standard deviations. Statistical significance (ns, *P* > 0.05; *, *P* ≤ 0.05; **, *P* ≤ 0.01; ***, *P* ≤ 0.001; ****, *P* ≤ 0.0001) was calculated for each condition versus “infected cells” by one-way ANOVA followed by Dunnett’s multiple comparisons test.

The binding of SARS-CoV-2 virions to cells is a crucial step in the replication cycle of SARS-CoV-2. The effect of lipopeptides on the binding of SARS-CoV-2 was evaluated by quantifying the viral RNA of virions bound to cells in different settings. In the first set of experiments, to investigate the interaction between the lipopeptides and the virus and evaluate its effect on binding, the virus was pre-treated with the lipopeptides before it was added to cells (named “pretreatment of SARS-CoV-2 followed by incubation on cells”). A second set of experiments to analyze the potential preventive effect of lipopeptides on cells consisted of treating the cells with the lipopeptides before the addition of the virus (called “pretreatment of cells followed by infection with SARS-CoV-2”). In the third set, the interaction between the lipopeptides and the cells was further investigated by removing unbound lipopeptides. In this experiment, the cells were incubated with the lipopeptides for 60 min and cleared from unbound lipopeptides before the addition of the virus (named “pre-infection treatment of cells”). Finally, the fourth set consisted of investigating the effect of lipopeptides on binding when the virus and the lipopeptides were added simultaneously to the cells (called “post-infection treatment of cells”) (Fig. 8).

When the virus was pre-treated, the neutralizing serum strongly reduced the percentage of SARS-CoV-2-bound cells, which reached 2% compared to the percentage of infected cells (no treatment). When the virus was pre-treated with lipopeptides, caspofungin did not show any effect on the binding percentage, while surfactin, WLIP, and fengycin all strongly reduced the binding percentage to 34.7%, 39.0%, and 21.7%, respectively. However, only the results obtained with surfactin and fengycin were statistically significant.

Pre-treating the cells with neutralizing serum was as efficient as when pre-treating the virus and was able to reduce the binding percentage of SARS-CoV-2 to the cells to 3.6% compared to infected cells. When lipopeptides were used to pre-treat the cells, all of them had a significant effect on the binding percentage of SARS-CoV-2. Surfactin, WLIP, and fengycin exhibited the greatest reductions in binding percentage, with binding percentages of 10.0%, 26.3%, and 11.3%, respectively. However, the cells pre-treated with caspofungin had a binding percentage of 81%.

When the cells were pre-treated with the lipopeptides and cleared from unbound lipopeptide before infection, only fengycin decreased the percentage of SARS-CoV-2-bound cells, with the percentage of bound cells divided by approximately two (54%).

Finally, when the lipopeptides were added simultaneously with the virus to the cells, none of the lipopeptides had a significant effect on reducing the binding percentage of SARS-CoV-2 to the cells.

#### Fusion inhibition

Several experiments were performed at our laboratory with relevant controls to determine the timing of the fusion of SARS-CoV-2 in Vero E6 cells (data not shown).

**Fig 9:**
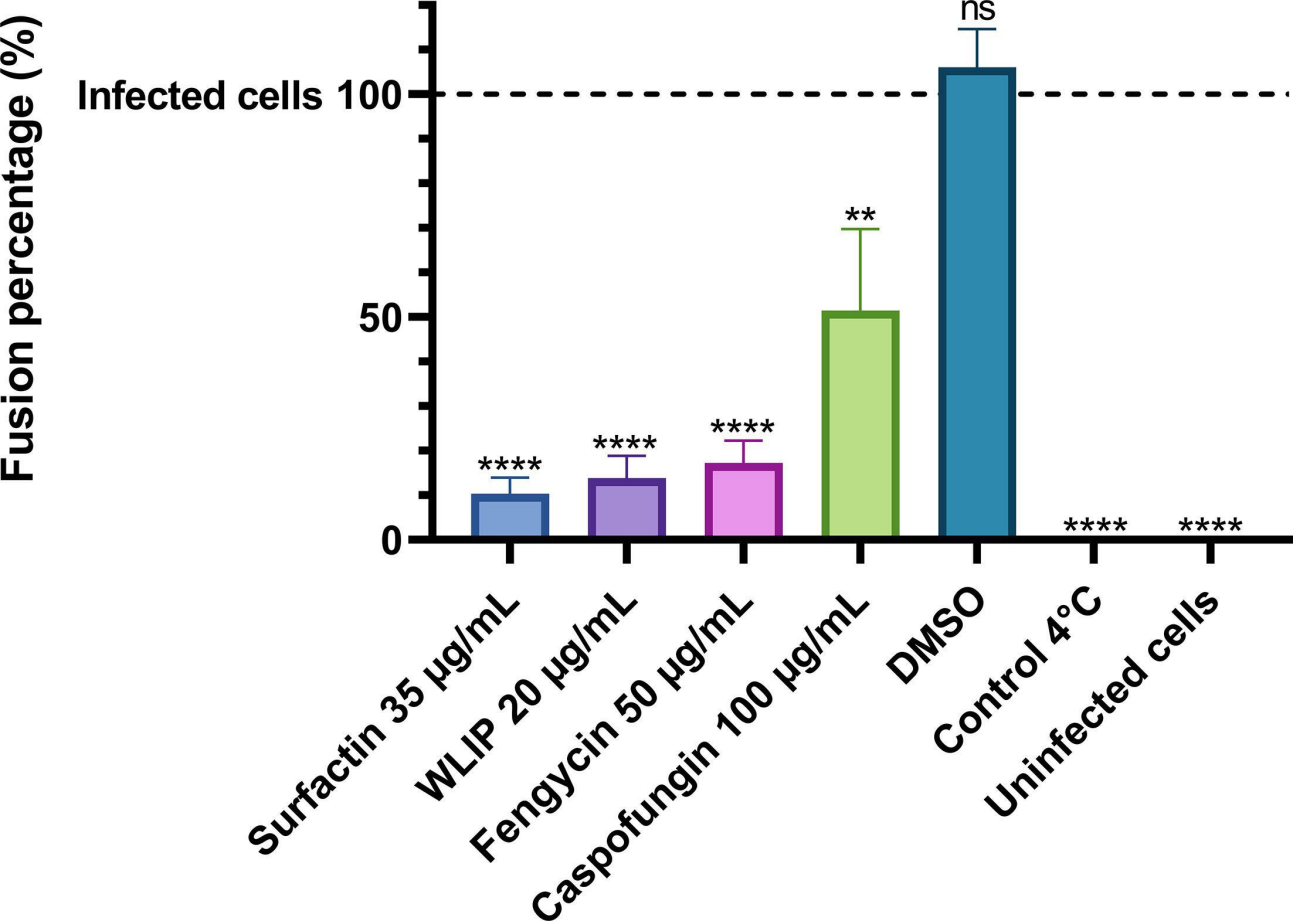
Surfactin, WLIP, fengycin, and caspofungin inhibited the fusion of SARS-CoV-2. The cells were infected on ice before removal of unbound virus and addition of the lipopeptides at 37°C to allow fusion of the virus with the cells. The values were normalized against those for infected cells (dashed line, no treatment) and the control (incubated at 4°C) and are plotted as the means of triplicate samples with standard deviations. Statistical significance (ns, *P* > 0.05; *, *P* ≤ 0.05; **, *P* ≤ 0.01; ***, *P* ≤ 0.001; ****, *P* ≤ 0.0001) was calculated for each condition versus “infected cells” by one-way ANOVA followed by Dunnett’s multiple comparisons test.

Fusion between the virus envelope and host cell membrane is another crucial step in the SARS-CoV-2 replication cycle. To investigate the potential inhibitory effect of the lipopeptides on this step, intracellular viral RNA was quantified after the binding of SARS-CoV-2 to the cells but before the fusion step, inhibited by rigidifying the cell membrane by working at low temperature. In that experiment, cells were infected on ice before removal of the inoculum, washing away unbound virus and adding lipopeptides while increasing the temperature to allow fusion of the virus with the cells. Two controls were used in this experiment. The positive control was untreated infected cells kept at 4°C, the temperature needed for fusion inhibition. The negative control consisted of untreated infected cells incubated at 37°C to allow fusion between the virions and the cells (100% fusion) (Fig. 9). Infected cells treated with lipopeptides showed a high reduction in fusion percentage, with all of them reducing the fusion to at least 51.4%. Surfactin, WLIP, and fengycin showed fusions reduced to 10.4%, 13.9%, and 17.2%, respectively. Caspofungin was the least effective lipopeptide for reducing the fusion between SARS-CoV-2 and the cells (fusion reduced to 51.4%).

#### Budding inhibition

Several experiments were performed at our laboratory with relevant controls to determine the timing of the budding of SARS-CoV-2 in Vero E6 cells (data not shown).

**Fig 10:**
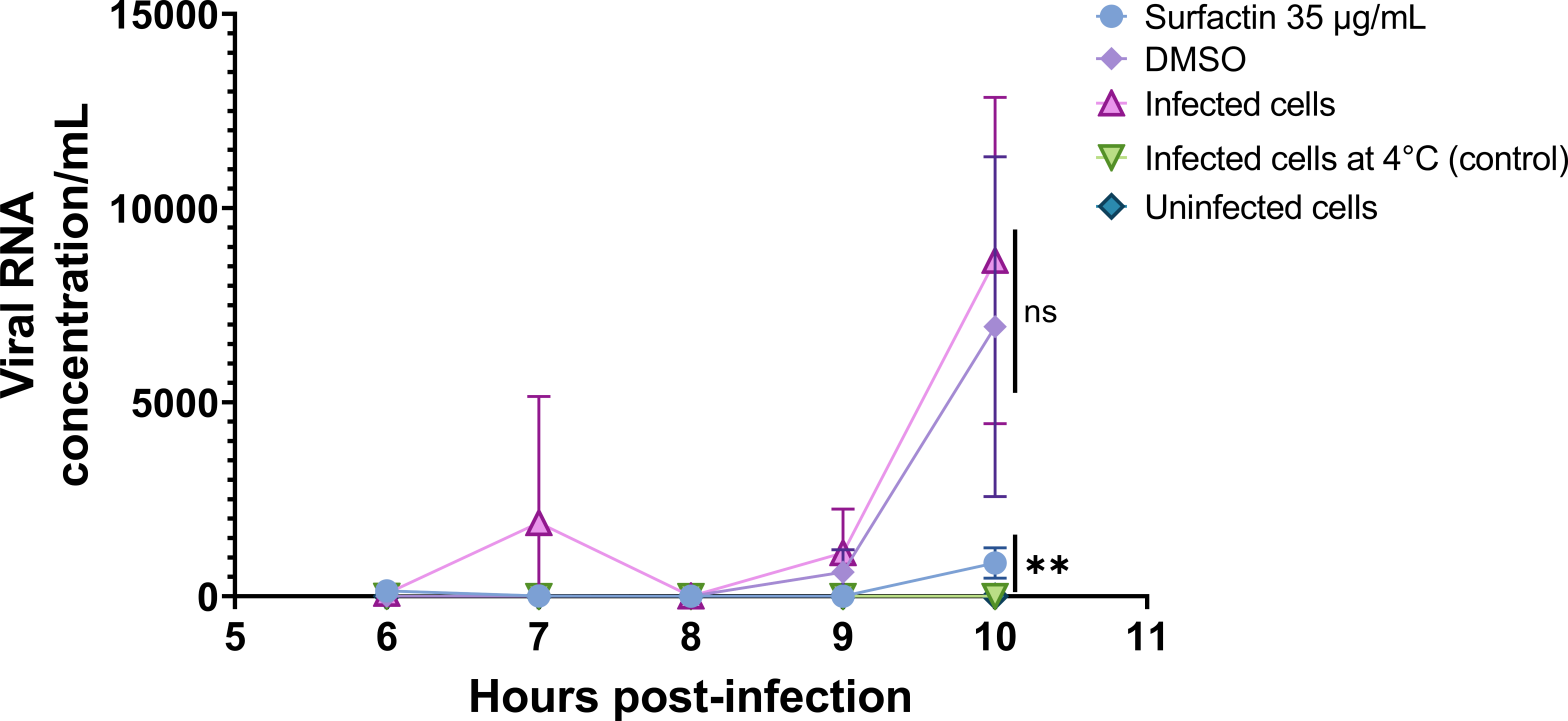
Inhibition of the budding of SARS-CoV-2 by surfactin, WLIP, fengycin and caspofungin. Cells were infected with SARS-CoV-2, after which the cells were washed to remove unbound virus, and the lipopeptides were added to the infected cells 6 h after infection. The release of virions in the supernatant was detected by RT‒qPCR. After infection, the control was kept at 4°C to inhibit the release of virions, and act as a positive control of the experiment. The values are plotted as the means of triplicate samples with standard deviations. Statistical significance (ns, *P* > 0.05; *, *P* ≤ 0.05; **, *P* ≤ 0.01; ***, *P* ≤ 0.001; ****, *P* ≤ 0.0001) was calculated for each condition versus “infected cells” by one-way ANOVA followed by Dunnett’s multiple comparisons test.

Budding is the final replication step involving membranes when the virion exits the host cell and acquires a host-derived membrane enriched in viral proteins to form the external envelope. The inhibition of the budding step by lipopeptides was assessed by infecting Vero cells with SARS-CoV-2, removing the inoculum and adding lipopeptides to the cells just before the release of new virions into the supernatant and by quantifying the RNA concentration per milliliter in the supernatant (Fig. 10). After infection, a control was kept at 4°C to rigidify the membranes and inhibit the release of virions (with an RNA concentration of 1.7 at 10 h pi). Of the four lipopeptides tested for their ability to inhibit budding, only surfactin decreased the RNA concentration per milliliter compared to that in infected cells (no treatment) from 8646.9 to 858.7 RNA concentration per milliliter, corresponding to a 1 log reduction at 10 h pi.

## DISCUSSION

Despite the treatments currently available for treating COVID-19, there is still a critical need for new antiviral molecules against SARS-CoV-2 that target different stages of the SARS-CoV-2 replication cycle. In our work, we classified more than 120 families of lipopeptides that have been described with various FA chain lengths, peptide moieties and compositions, structures, and charges. Some of these molecules have already been shown to exhibit antiviral effects, mostly against enveloped viruses (26–32), although their mechanism of action is still not fully understood, and their antiviral activity is usually not differentiated from their cytotoxicity. Thus, in our work, we selected 15 different lipopeptides from nine different families that presented different structures, FA chain lengths, peptide moieties and charges to better understand the structure-activity relationships of lipopeptides.

The screening revealed that 10 lipopeptides were cytotoxic at concentrations lower than 100 µg/mL and that this cytotoxicity was concentration dependent. Polymyxin B, colistin, daptomycin, and caspofungin did not cause any cytotoxicity at concentrations up to 100 µg/mL, which is in accordance with the results of other studies (47–53). As expected, in our cytotoxicity assay, fengycin exhibited lower cytotoxic activity than other lipopeptides from *Bacillus* sp., with an IC_50_ comparable to what was previously reported (54). Non-ionic lipopeptides were more cytotoxic than charged (anionic and cationic) lipopeptides, with all the non-ionic lipopeptides tested reaching 0% viability at 100 µg/mL. In contrast, the cationic lipopeptides were less cytotoxic than the anionic and non-ionic lipopeptides.

When screening the lipopeptides for their anti-SARS-CoV-2 activity, at the highest concentration tested, six lipopeptides were able to reduce the RNA concentration/mL of SARS-CoV-2 in infected cells to undetected levels (over 6-log reduction), while six others reduced the RNA concentration by 2.5 to 4.1-log, and three lipopeptides did not impact the RNA concentration. This reduction in the RNA concentration to below the level of residual viral RNA (SARS-CoV-2 at an MOI of 0.01 in medium without cells) at a lipopeptide concentration of 100 µg/mL could be explained by the cytotoxicity in these conditions and the potential release of RNases after cell lysis by the lipopeptides. However, while no cell viability was observed for iturin or mycosubtilin at that concentration, viral RNA was still detected. These findings suggest a potential direct antiviral effect of some other lipopeptides, especially those where viral RNA is undetectable. However, how these structural differences impact the antiviral effect of lipopeptides at cytotoxic concentrations remains to be elucidated.

Based on the PLSR analysis, the main structural traits positively correlated with the selectivity index were the number of charged amino acids, the number of amino acids and the negative charges at pH 7. In other words, the optimal lipopeptide would have a high number of amino acids, most of which would be anionic amino acids, to maximize the selectivity index. The four lipopeptides surfactin, fengycin, WLIP and caspofungin selected for their high antiviral potential and the in-depth investigation of their antiviral mechanism have a high number of amino acids, ranging from 6 (for caspofungin) to 10 (for fengycin). In addition, they all have charged amino acids, all of which contain negatively charged lipopeptides except caspofungin, which has positively charged amino acids.

**Fig 11:**
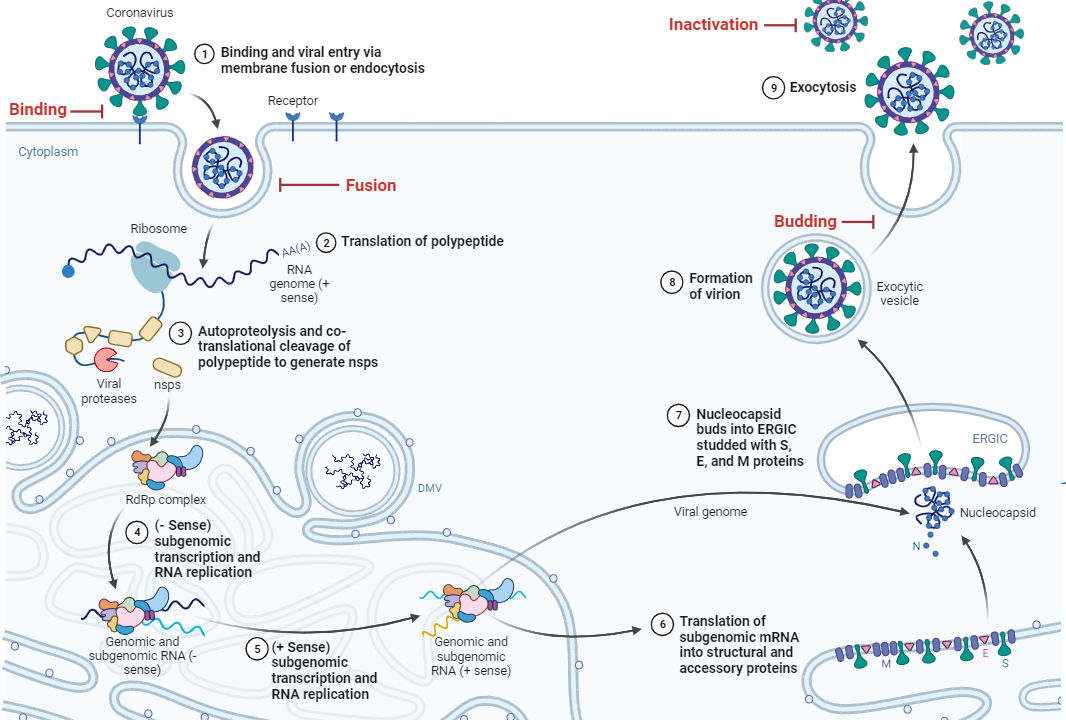
The reproductive cycle of SARS-CoV-2 and its replication stages during which it potentially interacts with lipopeptides. The highlighted steps in red represent the specific membrane-related stages that were investigated in our *in vitro* research. Figure adapted from (56).

Surfactin, which is one of the most studied lipopeptides, was one of the two lipopeptides associated with the greatest reduction in viral RNA in the viral replication assay. Surfactin at 35 µg/mL reduced the viral RNA concentration to levels similar to those of the neutralizing serum. In the replication inhibition assay, the results were significant at 24 h and 72 h pi but not at 48 h pi as the variability at 48 h pi was higher than at 24 h and 72 h pi, resulting in non-significant results. However, the tendencies observed at 24 h and 72 h pi can also be observed at 48 h pi. We confirmed that at the concentration tested (35 µg/mL), surfactin did not directly inactivate SARS-CoV-2; however, at a higher concentration (100 µg/mL), partial inactivation of the virus was observed, as shown for other coronaviruses (30). Surfactin at 35 µg/mL had an impact on the three steps involving the viral envelope. Surfactin strongly reduced the binding of SARS-CoV-2 when used to pre-treat the cells, inhibited the fusion step of the virus and was the only tested lipopeptide that affected the budding of the virus. The effect of surfactin on SARS-CoV-2 budding could be either a delay or an inhibition of the budding of SARS-CoV-2. The results obtained here suggest that surfactin has broad anti-SARS-CoV-2 activity, impacting the viral life cycle at different stages, which supports the hypothesis that the target of lipopeptides is thought to be membranes (i.e., the viral envelope and cellular membranes) (Fig. 11) (55).

WLIP belongs to the viscosin family, for which antiviral properties have been described (57). Among the chosen lipopeptides, WLIP exhibited the second-highest level of effectiveness, as indicated by the significantly reduced viral RNA levels in the viral replication assay. As for surfactin, WLIP at 20 µg/mL reduced the viral RNA concentration to levels similar to those of the neutralizing serum, but WLIP showed a slight direct inactivation of SARS-CoV-2 at this concentration. Cells pre-treated with WLIP showed a reduction in the binding of SARS-CoV-2, and WLIP also had an impact on the fusion of the virus. While WLIP significantly decreased viral RNA levels during the viral replication assay, our experiments suggest that its antiviral activity may be narrower in scope than that of surfactin.

Antiviral properties have also been reported for the fengycin family (58). In our work, compared with the other lipopeptides, fengycin at 50 µg/mL only slightly reduced the amount of viral RNA in the replication assay. However, pre-treatment with fengycin inhibited the binding of SARS-CoV-2 to cells. Interestingly, fengycin was the only lipopeptide that reduced viral binding after it was washed away. This could indicate an interaction between fengycin and the cells, which could inhibit the binding of the virus to the cells. In addition, fengycin also had an effect on viral fusion. Fengycin is the only tested lipopeptide that could have a preventive action against SARS-CoV-2.

Different antiviral properties have been described for lipopeptides belonging to the same family as caspofungin (37). Although caspofungin reduced the viral RNA concentration in the replication assay, the effect of caspofungin in the other assays was less important than that of other lipopeptides. Caspofungin only slightly reduced the binding of SARS-CoV-2 when pre-treating the cells and it was the least effective lipopeptide for reducing viral fusion. The effect of caspofungin on viral replication could be explained by other mechanisms that remain to be elucidated.

Taken together, these results show that despite having similar structures, some lipopeptides display promising antiviral activity, acting at different stages of virus replication, as generally illustrated in Fig. 11.

In our work, kidney epithelial cells were used, although SARS-CoV-2 is known to primarily target the lungs; thus, the cytotoxicity of lipopeptides against ciliated respiratory epithelial cells needs to be investigated. *In vivo* experiments have shown that lipopeptides can prevent the infection of piglets by PEDV (31), but the cytotoxic and antiviral activities of lipopeptides in animals remain to be studied. In addition, we tested pure lipopeptides to determine their specific mode of action against SARS-CoV-2. However, lipopeptides are usually produced as a mixture by bacteria and are known to act in a synergistic manner. For example, surfactin increases the hemolytic activity of iturin A (59). A more recent study revealed that a mixture of surfactin and fengycin is effective against a strain of *Venturia inaequalis,* while both molecules are ineffective alone (60, 61). This synergy should be investigated for the cytotoxic and antiviral activities of lipopeptides. Combining lipopeptides acting at different steps of virus replication could lead to more efficient SARS-CoV-2 treatment.

For the purpose of this work, we selected only 15 different lipopeptides and further studied only four different molecules. There is a very high diversity of lipopeptides and different modes of action that should be further explored. Finally, as we hypothesize that lipopeptides impact the viral reproductive cycle involving membranes, further studies should be conducted to investigate the impact of the membrane composition on the effect of lipopeptides, as the membranes involved in different viral reproductive cycle steps (i.e., fusion or budding) have different lipidic compositions. *In vitro* studies should be complemented with mechanistic studies such as the one conducted by Shekunov et al. (38) to further understand the antiviral effects of lipopeptides. They conducted *in vitro* studies on Vero cells to assess the impact of lipopeptides. Among the tested lipopeptides, aculeacin A, anidulafugin, iturin A (with an IC_50_ of 136 µg/mL), and mycosubtilin (with an IC_50_ of 115 µg/mL) were identified as effective at reducing the cytopathogenicity of SARS-CoV-2 without causing specific toxicity. However, our study revealed that iturin A and mycosubtilin (with IC_50_ values of 4.9 and 1.8 µg/mL, respectively) are among the most cytotoxic lipopeptides we tested, with no distinguishable antiviral activity from their cytotoxic effects. Furthermore, they indicated that surfactin, daptomycin, and polymyxin B exhibit high cytotoxicity (with IC_50_ values of 0.6, 0.7 and 1.1 µg/mL, respectively), contradicting our results (with an IC_50_ of 51.5 µg/mL for surfactin and over 100 µg/mL for daptomycin and polymyxin B), as well as findings discussed earlier. When working with lipopeptides, the focus should be on solubilization, and it is crucial to confirm and quantify them after solubilization. Shekunov et al. mentioned solubilizing their lipopeptides in DMSO but did not specify concentrations for the lipopeptide stocks or provide information on how they confirmed complete solubilization.

This study highlights the fact that lipopeptides, although sharing similar structural features, can have variable effects as soon as one of these parameters is modified. To better understand these mechanisms of action, it appears necessary to carry out biophysical studies on simplified systems, which will allow a better description and understanding of the specifics of these molecules. The combination of *in vitro* studies with mechanistic studies such as the one conducted by Shekunov et al. (38) or with studies such as the one presented here will therefore allow a deeper understanding of the antiviral effects of lipopeptides.

### Conclusion

Our work aimed at better understanding the antiviral activities of lipopeptides and provide insight into their structure-activity relationships. Among the 15 lipopeptides tested, surfactin, WLIP, fengycin and caspofungin were the most promising lipopeptides. These four lipopeptides had an impact on all the steps involving the viral envelope. Surfactin and WLIP are the two lipopeptides that showed the greatest reduction in viral RNA in the replication assay, comparable to neutralizing serum. Surfactin was the only lipopeptide that had an impact on the budding of SARS-CoV-2, while fengycin was the only lipopeptide that impacted the binding of SARS-CoV-2 after pre-infection treatment of the cells. Our results highlighted the principal structural traits of lipopeptides impacting the selectivity index, and this ideal lipopeptide would have a high number of amino acids with a high number of charged (and more specifically anionic) amino acids. Our work was the first step in the design of new lipopeptides that exhibit low cytotoxicity and high antiviral activity, potentially leading to the development of effective treatments.

## Data Availability

The data sets used and/or analyzed during the current study are available from the corresponding author on reasonable request.

## SUPPLEMENTAL MATERIAL

Table S1 aem.01036-24-s0001.xlsx The 120 lipopeptide families meeting our criteria as lipopeptide.

ASM does not own the copyrights to Supplemental Material that may be linked to, or accessed through, an article. The authors have granted ASM a non-exclusive, world-wide license to publish the Supplemental Material files. Please contact the corresponding author directly for reuse.
